# Mapping propagation of collective modes in Bi_2_Se_3_ and Bi_2_Te_2.2_Se_0.8_ topological insulators by near-field terahertz nanoscopy

**DOI:** 10.1038/s41467-021-26831-6

**Published:** 2021-11-18

**Authors:** Eva Arianna Aurelia Pogna, Leonardo Viti, Antonio Politano, Massimo Brambilla, Gaetano Scamarcio, Miriam Serena Vitiello

**Affiliations:** 1 NEST, CNR-Istituto Nanoscienze and Scuola Normale Superiore, Piazza San Silvestro 12, 56127 Pisa, Italy; 2 Department of Physical and Chemical Sciences, University of L’Aquila, via Vetoio 1, 67100 L’Aquila, Italy; 3 Dipartimento Interateneo di Fisica, Università degli Studi e Politecnico di Bari, and CNR-Istituto di Fotonica e Nanotecnologie, via Amendola 173, 70126 Bari, Italy

**Keywords:** Topological insulators, Electronic properties and materials

## Abstract

Near-field microscopy discloses a peculiar potential to explore novel quantum state of matter at the nanoscale, providing an intriguing playground to investigate, locally, carrier dynamics or propagation of photoexcited modes as plasmons, phonons, plasmon-polaritons or phonon-polaritons. Here, we exploit a combination of hyperspectral time domain spectroscopy nano-imaging and detectorless scattering near-field optical microscopy, at multiple terahertz frequencies, to explore the rich physics of layered topological insulators as Bi_2_Se_3_ and Bi_2_Te_2.2_Se_0.8_, hyperbolic materials with topologically protected surface states. By mapping the near-field scattering signal from a set of thin flakes of Bi_2_Se_3_ and Bi_2_Te_2.2_Se_0.8_ of various thicknesses, we shed light on the nature of the collective modes dominating their optical response in the 2-3 THz range. We capture snapshots of the activation of transverse and longitudinal optical phonons and reveal the propagation of sub-diffractional hyperbolic phonon-polariton modes influenced by the Dirac plasmons arising from the topological surface states and of bulk plasmons, prospecting new research directions in plasmonics, tailored nanophotonics, spintronics and quantum technologies.

## Abstract (web summary)

Propagation of photoexcited modes provides important information for novel quantum state. Here, the authors reveal the propagation of sub-diffractional hyperbolic phonon-polariton modes influenced by the Dirac plasmons in topological insulators.

## Introduction

Rhombohedral layered compounds of bismuth with group VI elements as Bi_2_Se_3_, Bi_2_Te_3_ and their alloys have attracted renewed interest as prime candidates for the development of photonic devices exploiting their topological nature,^1,2^ thanks to their thickness-dependent bandgap and to the electronic dispersion of their topological surface states (TSS)^3^. These topological insulators (TIs)^1^ are semiconductors in the bulk, exhibiting metallic conduction at the surface, activated by the TSS, which form a Dirac cone around the Γ point of a hexagonal Brillouin zone^2^.

TIs provide a versatile platform for investigating quantum phenomena and for applications in electronics and photonics: with almost the same high-absorbance of graphene^4^, they can exploit a tunable surface bandgap^5^; their optical and electronic properties can be engineered by changing the material stoichiometry^6^; and the two-dimensional electron gas (2DEG) arising from the TSS can support collective excitation (Dirac plasmon) at terahertz (THz) frequencies^7,8^. This opens intriguing perspectives for a variety of application fields, such as quantum computing^9^, spintronics^10^, and photodetection^6^.

Some of these applications may benefit from the exploitation of electronic collective excitations, like plasmons,^11,12^ plasmon-polaritons^13^—hybrid light–matter modes involving the collective oscillations of charge carriers, phonons^14^ or phonon-polaritons^15,16^, whose wavelength can be electrically controlled. However, capturing these modes on the TSS of a TI or even in the bulk is a very demanding task: it requires sophisticated optical techniques capable of imaging at their exact frequency or mapping their long-range propagation; very importantly, the correct identification of TSS requires the knowledge of their complete energy/momentum dispersion, that can be only unveiled in TI samples showing sufficiently high structural quality (both stoichiometric and crystallographic)^17,18^.

Amplitude- and phase-resolved scattering near-field optical microscopy (s-SNOM)^14,15,19–25^ can allow access to the spatial variation of complex-valued dielectric response of layered 2D materials, heterostructures and low dimensional systems, therefore providing the sub-diffractional spatial resolution required to investigate light–matter interaction at the nanoscale. Moreover, s-SNOM is the ideal tool to investigate polaritons since its sharp tip acts both as a launcher and as a detector of propagating collective modes^15^. A large variety of interferometric approaches has been developed in the last years, allowing amplitude and phase-resolved s-SNOM imaging in the visible and infrared spectral ranges^19,20^, including fs pulsed laser sources and electro-optic sampling detection in the THz range,^21^ microwave circuitry in the sub-THz range^22,23^ or interferometric techniques^24,25^ strongly limited by the poor dynamic range of the cryogenically cooled bolometric detectors needed to measure the typically small s-SNOM THz signals.

At THz frequencies, the dielectric response of TIs based on bismuth chalcogenides is characterized by longitudinal (LO) and transverse (TO) optical phonons that identify frequency regions of hyperbolicity, within which the dielectric permittivities along the in-plane and out-of-plane directions have an opposite sign and the isofrequency surfaces of the extraordinary rays are hyperboloids in the momentum space^26,27^. Hyperbolic phonon-polaritons modes have been intensively investigated in hexagonal boron nitride (hBN)^15,28,29^, where they occur at mid-infrared frequencies, due to their unique capability to support the propagation of light with momentum far exceeding the free-space value without evanescent decay^15,28^. Remarkably, in TIs, these polaritons, that have great potential for THz waveguiding, can interact with the electrons associated to the TSS resulting in doping tunable dispersion^30^.

In this letter, we exploit an innovative combination of hyperspectral time-domain spectroscopy (TDS) nano-imaging^31^ and detectorless scattering near-field optical microscopy (s-SNOM)^14^, at three distinctive pumping energies in the far-infrared, to explore the rich physics of thin flakes of Bi_2_Se_3_ and Bi_2_Te_2.2_Se_0.8_, having different thicknesses. We select Bi_2_Se_3_ because of its simple electronic structure, showing a topologically nontrivial direct energy gap of 0.3 eV^32,33^, about ten times higher than thermal excitation at RT^34^ and higher than that of Bi_2_Te_3_, having an energy gap of about 0.15 eV^35^. We then investigate Bi_2_Te_(3−3x)_Se_3x_ because, on one side, the energy gap is expected to increase rapidly with x, reaching values >0.2 eV for 3x = 0.8^35^, and on the other side, the Fermi level of high Te-content alloys, like Bi_2_Te_2.2_Se_0.8_, crosses only the TSS Dirac cone, meaning that its TSS electrons behave as an ideal 2D electron gas^36^.

Theoretical proposals^2^ on TSS require the chemical potential to lie at, or to approach, the surface Dirac point. However, the surface Fermi level of a TI depends on the detailed electrostatics of the surface and it is not necessarily at the Dirac point. In a naturally grown Bi_2_Se_3_, the bulk Fermi energy does not lie in the gap^33^. The material displays n-type behavior and usually exhibits significant contributions from the bulk carriers to the total conductivity^33,34,37–39^. One reason for the shift of the chemical potential into the conduction band is donor doping, typically induced by Se vacancies^2^ and antisite defects^40^. Another effect, which can interfere with the ideal TI response, occurs when the bulk bands cross the Fermi level near the material surface, generating a 2DEG of massive carriers close to the surface if the bands bend downwards^41^. This has been observed in similarly grown single-crystal Bi_2_Se_3_ when exposed to the atmosphere after cleaving^42^, as a consequence of extrinsic defects or molecular adsorption at the surface^36^. The 2DEG is believed to be confined within 20 nm from the surface in Bi_2_Se_3_^36^ and coexists with the TSS. Engineering the Bi_2_Te_(3−3x)_Se_3x_ stoichiometry can therefore allow tuning the TI physics, on purpose.

Our combination of two different near-field techniques allows identifying optically activated TO and LO phonons, and reveals massive bulk plasmons, arising from the 2DEG originated from the band bending at the sample surfaces in Bi_2_Se_3_, and hybrid plasmon–phonon-polariton modes propagating from the edge of the Bi_2_Te_2.2_Se_0.8_ flakes. Remarkably, this represents the first experimental demonstration of hybrid plasmon–phonon-polariton modes reported to date in topological insulators and paves the way for novel sub-diffractional and tunable THz polaritonic waveguides.

## Results and discussion

### Samples

Flakes of thickness d in the range 15–200 nm and lateral size in the range 0.2–4 μm are obtained by mechanical exfoliation on a 350-μm-thick silicon wafer, with a 300 nm thin insulating SiO_2_ top-layer. The micro Raman characterization of prototypical flakes of Bi_2_Se_3_ and Bi_2_Te_2.2_Se_0.8_ is reported in Supplementary Fig. 1a, b, respectively. Bi_2_Se_3_ and Bi_2_Te_3_ crystallize in tetradymites with rhombohedral crystal structure endowed with D^5^_3d_ space group symmetry and five atoms per unit cell. The structure consists of quintuple layers (QL) stacked via weak van der Waals forces, which permit an easy mechanical exfoliation, orthogonally to the trigonal c-axis. One QL corresponds to a 1.42 nm thick crystal of Bi_2_Se_3_ and to 1 nm for Bi_2_Te_2.2_Se_0.8_^6^. Within each QL, pictogen (Bi) and chalcogen atoms (Se, Te) are held together by ionic covalent bonds. The different bond strength and character result in a strong in-plane/out-of-plane anisotropy in the thermal, electronic and dielectric properties of these materials.

### Time-domain near-field spectroscopy: the role of hyperbolic phonons

**Fig. 1:**
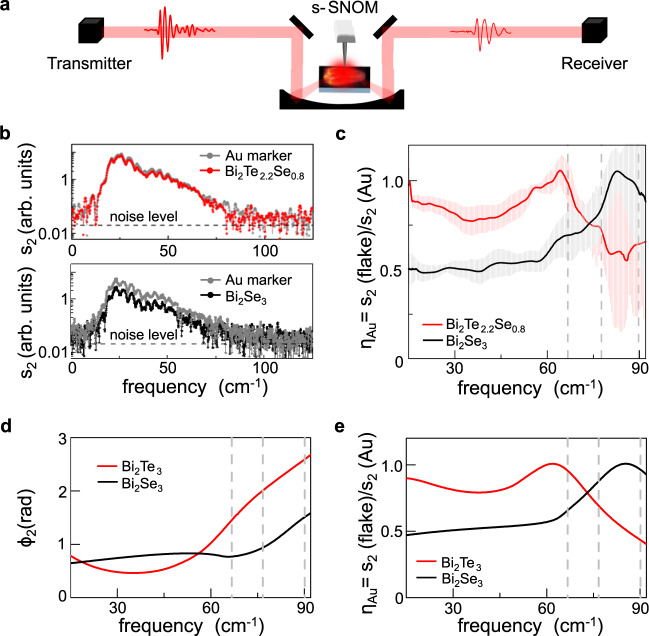
Hyperspectral THz nano-imaging of TI. **a** Sketch of the hyperspectral THz nano-imaging experiment based on detection of the THz field forward scattered by the AFM tip of the s-SNOM with a TDS system based on two photoconductive antennas: the receiver and the transmitter. **b** Spectra of second-order demodulated signal s_2_ scattered by thick flakes of Bi_2_Se_3_ (d = 86 nm, lower panel, black circles and line) and Bi_2_Te_2.2_Se_0.8_ flakes (d = 76 nm, upper panel, red circles and line) as compared to the scattered spectra from a 140 nm thick Au reference marker (gray circles and line). **c** Contrast η_Au_ evaluated dividing the average of 60 spectra s_2_ scattered by Bi_2_Te_2.2_Se_0.8_ (red line) and Bi_2_Se_3_ (black line) by the average of 60 spectra measured on Au reference markers placed near the flakes after smoothing each curve with the average on 20 points window. The error bars are evaluated as standard deviation. **d**, **e** Near-field phase ϕ_2_ (**d**) and contrast η_Au_ (**e**) of the second-order demodulated signal s_2_ as a function of frequency for bulk Bi_2_Se_3_ (black line) and Bi_2_Te_3_ (red line) retrieved via numerical inversion^51^ of the finite-dipole model on a four-layer structure (see Supplementary Fig. 6). Gray dashed lines in panels c–e mark the probing frequencies of the detectorless near-field experiment.

Hyperspectral THz nano-imaging^31^ is performed by coupling the s-SNOM microscope to a THz TDS system based on photoconductive (PC) antennas (Menlo TERA15 FC), as schematically depicted in Fig. 1a.

The broadband THz pulse produced by the transmitter antenna, with a spectrum spanning the 4–140 cm^−1^ range, is focused on the atomic force microscope (AFM) tip of the s-SNOM and the forward-scattered field is detected as a function of the time delay from the gate pulse by a second PC antenna, the receiver, working in reverse mode. This technique combines broadband spectral coverage with sub-diffractional spatial resolution of ~260 nm (see Supplementary Fig. 4) achieved by using an AFM tip with apex radius *r* = 40 nm, 80 μm cantilever length (Rocky Mountain Nanotechnology, 25PtIr300B) working in tapping mode, with an oscillation amplitude A ~200 nm and a tapping frequency Ω ~ 20 kHz.

The detected THz scattered field *S*_n_ = *s*_n_ e^−i*ϕ*^, with amplitude s_n_ and phase ϕ, is demodulated at the tapping frequency *n*th harmonics Ω_*n*_ = *n*Ω with *n* = 2–4 to isolate the near-field contribution from the background due to far-field illumination of the tip shaft and sample^19^. Images of the near-field scattered amplitude s_n_, at a fixed time delay, for thick flakes (d > 75 nm) of Bi_2_Se_3_ and Bi_2_Te_2.2_Se_0.8_ as a function of the demodulation order n, are reported in Supplementary Fig. 2a, b. To find a good compromise between signal intensity and suppression of the far-field background, we investigate the second harmonic near-field signal. This choice is supported by the analysis of the approach curves (Supplementary Fig. 3) at the different demodulation orders.

Nano-THz spectra at fixed positions on thick (d > 75 nm) Bi_2_Se_3_ and Bi_2_Te_2.2_Se_0.8_ flakes and on a reference point on an Au marker are reported in Fig. 1b. Each spectrum is obtained by averaging 60 different spectra acquired with 100 ps time-scan, lasting 45 s, performed in a nitrogen atmosphere to prevent absorption by water vapor^43^. Due to the frequency-dependent scattering efficiency of the tip^44^, a signal-to-noise ratio (SNR) > 1.5 is achieved only in the range 15–90 cm^−1^. In this range, the two crystals exhibit distinct contrast η_Au_, evaluated by the ratio of the second-order demodulated spectra s_2_ scattered by the flake with that of the Au reference, as routinely done in nano-FTIR spectroscopy^45^. While both compounds show a broad peak in the probed range, the maximum η_Au_ is reached at ~80 cm^−1^ for Bi_2_Se_3_ and at ~60 cm^−1^ for Bi_2_Te_2.2_Se_0.8_. This scattering enhancement can be attributed to infrared-active bulk optical phonons^46–48^. The peak in the near-field scattering intensity of Bi_2_Se_3_ occurs within the frequency band ω_to_^⊥^ < ω < ω_to_^//^ identified by the dominant long-wavelength TO optical phonon modes^30^: the E_u_ TO (ω_to_^⊥^ = 64 cm^−1^) and the A_2u_ TO (ω_to_^//^ = 135 cm^−1^) phonons, involving atomic vibrations in the plane orthogonal (⊥) and parallel (//) to the trigonal c-axis, respectively. Within this frequency range, the real parts of the permittivity along the directions orthogonal ε^⊥^ and parallel ε^//^ to the c-axis have an opposite sign (Re{ε^⊥^} < 0, Re{ε^//^}>0) and Bi_2_Se_3_ is expected to behave as a hyperbolic material of type II. We can use the vibrational modes of Bi_2_Te_3_ to model the Bi_2_Te_2.2_Se_0.8_ flake, due to the low dispersion^46^ of the phonon modes of the Bi_2_Te_(3−3x)_Se_3x_ alloy for x < 1/3. The lower frequency of the Bi_2_Te_2.2_Se_0.8_ peak can be traced back into the red-shift of E_u_ and A_2u_ TO phonons with increasing Te-content towards the values reported for Bi_2_Te_3_ (ω_to_^⊥^ = 50 cm^−1^ and ω_to_^//^ = 95 cm^−1^)^46,47^.

Three-dimensional model calculations of the near-field contrast η based on the well-established finite-dipole approximation^49,50^, considering multiple LO and TO phonon modes, both in // and ⊥ directions, are reported in the Supplementary Fig. 5a. The model well reproduces the experimental spectra, suggesting that Bi_2_Te_3_ can reasonably mimic the near-field response of the Bi_2_Te_2.2_Se_0.8_. Moreover, since the exposed surfaces are parallel to the basal plane, the ability of detecting ⊥ modes implies that the component of the THz near-field orthogonal to the tip-tapping is non-negligible, as previously observed in anisotropic crystals as SiC^49^.

In order to disentangle the dielectric response from the bulk and the surface, we then combine the finite-dipole approach to describe the tip-sample interaction, with a multilayer model for up to four layers and we implement an inversion algorithm to extract the local dielectric function of the surface layer alone^51^ (see Supplementary Note 3 and Fig. 6). We note that even if we include a finite bulk carrier density, both Bi_2_Se_3_ and Bi_2_Te_2.2_Se_0.8_ maintain their hyperbolic behavior as testified by the opposite signs of the real parts of the bulk dielectric functions in the // and ⊥ directions (see Supplementary Fig. 6d, e). The comparison between the two models (the multilayer model in Fig. 1e and the bulk model in Supplementary Fig. 5a) clearly unveils that while in Bi_2_Se_3_ the amplitude contrast is well reproduced by the bulk model and the TSS do not appear to contribute to the near-field response (black traces in Fig. 1e and Supplementary Fig. 5a), to describe the low frequency (ω < 50 cm^−1^) amplitude contrast measured in Bi_2_Te_2.2_Se_0.8_, we have to include a finite contribution from TSS to the near-field amplitude (see red traces in Fig. 1e and Supplementary Fig. 5a).

### Detectorless near-field nanoscopy: role of the topological surface states

**Fig. 2:**
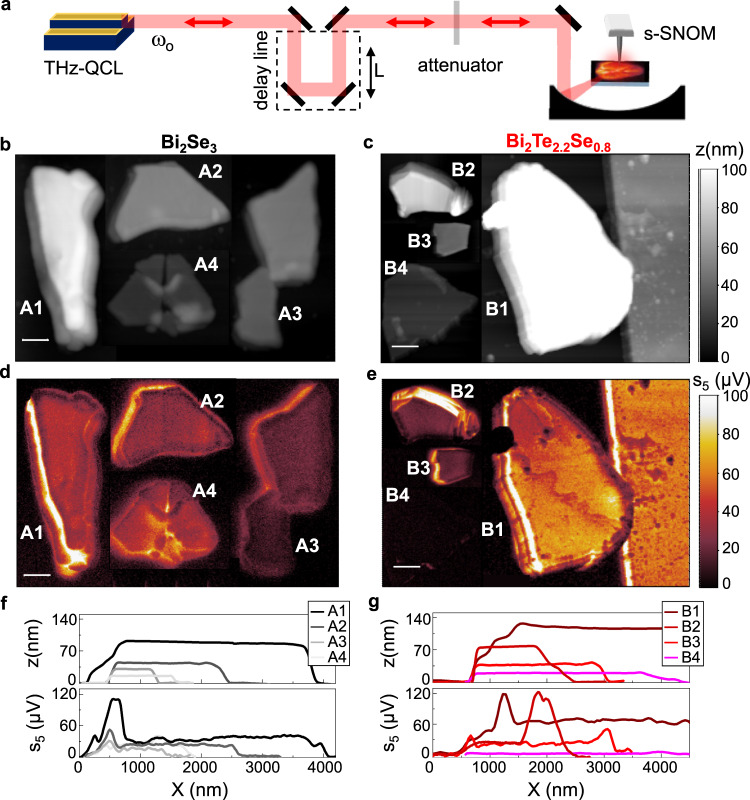
Self-detection THz nanoscopy of TIs. **a** Sketch of the self-detection nanoscopy experiment based on THz-QCL of frequency ω_0_ = 66.7, 76.7, 89.7 cm^−1^ simultaneously serving as source and detector of the backscattered field from the AFM tip of the s-SNOM with a delay-line controlling the length L of the optical path and an attenuator to reduce the feedback intensity. **b**, **c** Topography maps of four distinctive flakes of Bi_2_Se_3_ (**a**) and Bi_2_Te_2.2_Se_0.8_ (**b**) of variable thickness z with labels Ai and Bi, respectively, with i = 1, 2, 3, 4. **d**, **e** Near-field maps of the fifth-order self-mixing signal s_5_ of the flakes in panels (**b**, **c**) taken while keeping a fixed external cavity length L, measured with ω_0_ = 66.7 cm^−1^ without attenuation. Scale bars in panels (**b**–**e**) correspond to 1 μm. **f**, **g** Line scans of the topography maps z and of the fifth-order self-mixing signal s_5_ on four distinctive flakes of Bi_2_Se_3_ (**f**) and of Bi_2_Te_2.2_Se_0.8_ (**g**), extracted from panels (**b**, **c**) and (**d**, **e**) while moving from the substrate to the flakes along a line orthogonal to the flakes edge.

With the aim of isolating the nature of the modes activated through THz photoexcitation and their interaction with TSS, we then perform self-detection nanoscopy on a set of flakes with a progressively reduced thickness, employing single-frequency mode THz-quantum cascade lasers (QCLs),^14^ as sketched in Fig. 2a, operating at three different emission frequencies, ω_0_ = 66.7, 76.7, 89.7 cm^−1^, corresponding to the gray dashed lines in Fig. 1c–e. THz-QCLs have the inherent advantage to provide mW output powers and high spectral purity, with intrinsic linewidths as low as 100 Hz^52^; this allows for selective interaction with THz resonant modes. In our self-detection experiment^14^, the QCL acts simultaneously as a powerful THz source and as a phase-sensitive detector, employing a detection scheme based on self-mixing (SM) interferometry^53^, therefore overcoming the need for bulky cryogenic detectors. This effect is based on the reinjection of a small fraction (10^−4^−10^−2^) of the emitted field that coherently interferes within the laser cavity. The fraction of the laser radiation backscattered from the tip-sample system can be coupled back into the QCL cavity along the same optical path. The coherent superposition of reinjected THz field with the QCL intracavity field produces a perturbation of the laser voltage ΔV that depends on both the amplitude and the phase of the THz field scattered by the tip.

s-SNOM experiments allow to excite collective modes of finite momentum by measuring the local response, which is given by the superposition of different modes with a distribution of momenta extended up to q $\sim$ 1/r, rather than by a single-mode. Exploiting the higher power and sensitivity of the self-detection experiment with respect to the TDS system, we use a tip with a smaller radius, *r* = 10 nm, corresponding to a spatial resolution of about 30 nm (see Supplementary Fig. 8) and to a larger probed momentum range.

In order to sample the SM fringes and retrieve the phase shift experienced by the field in the scattering process, the length L of the external cavity, formed by the tip and output laser facet, is varied up to 350 μm (>2λ = 4πc/ω_0_) with a delay line built on a linear translation stage with sub-μm spatial resolution (Fig. 2a).

A variable attenuator with transmission t is inserted in the optical path to reduce the optical feedback to the weak limit in which ΔV has a simple sinusoidal dependence on L, expressed, within the framework of the Lang–Kobayashi model^54^, as $\varDelta V \sim s{{{\rm{cos }}}}(\frac{{2\omega }_{0}L}{c}-\phi )$, with s signal amplitude and ${{{\rm{\phi }}}}$ phase of the scattered field. Lock-in detection of the *n*th harmonics of the tapping frequency Ω is then used to isolate the near-field component from the scattering background. Using a lock-in detection technique we have a noise level of 0.1 μV and a SNR > 40 on the *n* = 5 harmonics on thick flakes.

The scattering signal at the highest harmonic order (*n* = 5), acquired keeping fixed the cavity length L, is enhanced at the flakes compared to the substrate, see Fig. 2d, e. For thicker flakes with d > 40 nm, the amplitude of s_5_ appears higher for Bi_2_Te_2.2_Se_0.8_ (B1-B2 in Fig. 2c) than for Bi_2_Se_3_ (A1-A2 in Fig. 2b) in agreement with the η_Au_ values extracted via hyperspectral THz nano-imaging (Fig. 1c). However, the two materials show a different dependence on thickness: while negligible amplitude variation is observed upon reducing the thickness of Bi_2_Se_3_ from 86 nm (flake A1) to 16 nm (A4) (see Fig. 2d), the s_5_ amplitude drops in Bi_2_Te_2.2_Se_0.8_ such that the 19 nm thick flake (B4) is barely distinguishable from the substrate (Fig. 2e).

We then extract line profiles from the maps in Fig. 2b, e while moving along a line orthogonal to the flakes edge and keeping fixed the cavity length L, to show the variation of the substrate/flake contrast with thickness in the two material systems (Fig. 2f, g).

**Fig. 3:**
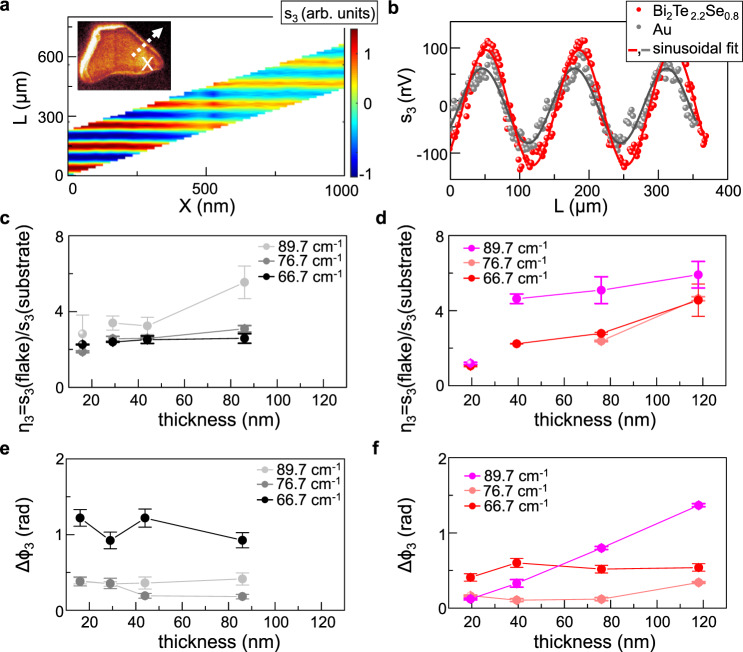
Amplitude and phase contrasts. **a** SM maps of s_3_ signal on flake A2 of Bi_2_Se_3_ as a function of the cavity length L and of the position X moving on the line orthogonal to the flake edge sketched in the inset by the white dashed arrow; the L sampling interval changes with X due to the delay line backlash. **b** SM fringes of s_3_ as a function of the cavity length L at a fixed position X on flake B3 of Bi_2_Te_2.2_Se_0.8_ (red dots) and on the substrate (gray dots) acquired at 76.7 cm^−1^ together with the sinusoidal best fit (solid lines). **c**, **d** Signal contrast η_3_ evaluated dividing the signal amplitude s_3_ on Bi_2_Se_3_ (**c**) and on Bi_2_Te_2.2_Se_0.8_ (**d**) by that collected on the substrate, all extracted from the sinusoidal fit of the SM fringes acquired on 1-μm-length X line scan across the flake/substrate interface, measured at three different probing frequencies. Error bars indicate 95% confidence bands from the fit. **e**, **f** Phase variation ${\triangle {{{\rm{\phi }}}}}_{3}$ from the substrate to the flakes, extracted from the sinusoidal fit of the SM fringes acquired on 1-μm-length X line scan across the flake/substrate interface, as a function of the flake thickness at the three different frequencies for Bi_2_Se_3_ (**e**) and Bi_2_Te_2.2_Se_0.8_ (**f**). Error bars indicate 95% confidence bands from the fit.

The thickness-dependent signal change, revealed in the line profiles (Fig. 2f, g), could be caused by both a phase shift and by an actual variation of the amplitude of the SM fringes. To discriminate between the two effects, we resolve the phase of the scattered field (predicted in Fig. 1d): we acquire the SM fringes with 0.2 μm steps while scanning the sample along a line orthogonal to the substrate-flake edge. Figure 3a shows the retrieved amplitude signal at the third demodulation order s_3_, as a function of L and of the position on the sample labeled as X.

Considering the drop in the scattering efficiency of the tip with increasing THz frequencies, we choose the third-order demodulated signal s_3_ to compare the data collected at the three different frequencies ω_0_. By analyzing the SM fringes at each X with sinusoidal fit functions (as in Fig. 3b), we extract the amplitude s_3_ and the phase ϕ_3_ along the scan trajectory (see Supplementary Fig. 9 for the phase profiles).

Since the absolute value of the SM signal depends on the threshold voltage of the THz-QCL, on its output power, and on the frequency-dependent scattering efficiency of the tip, the average scattering signal amplitude from the flake s_3_(flake), is divided by the signal from the substrate, s_3_(substrate), whose response is expected to show negligible variation with frequency. The refractive index variation of SiO_2_ in the 60–90 cm^−1^ range is indeed <0.5%^55^. The thickness dependencies of the ratio η_3,_ which is accounting for the contrast between flake and substrate, and of the phase change Δϕ_3_, at the three pumping frequencies, are reported in Fig. 3c, e, and in Fig. 3d, f, for Bi_2_Se_3_ and Bi_2_Te_2.2_Se_0.8_, respectively. The near-field response appears very different in the two materials.

In Bi_2_Se_3_, the flat topography regions show roughly constant contrast with the SiO_2_ substrate changing the flake thickness d, at all the probing frequencies, while only the signal at the flake edges varies with thickness. Looking at s_3_(ω_0_), by keeping fixed the layer thickness, the contrast η_3_ increases by 30–40% while increasing ω_0_ from ω_0_ = 66.7 cm^−1^ to ω_0_ = 76.7 cm^−1^ in Bi_2_Se_3_ (Fig. 3c). This is in disagreement with the calculated 2D near-field response of Bi_2_Se_3_ that predicts^30^ a peak in the s_3_ amplitude at the TO phonon wavenumber ω_to_^⊥^ = 64 cm^−1^ and a strong decrease of about 40% in the range 65–90 cm^−1^. On the other hand, the observed increase of *s*_3_ is compatible with the overall increase in the same range shown by the calculated contrast in Fig. 1e, and hence suggests a bulk phonon-like nature for the dielectric response of our Bi_2_Se_3_ samples, in agreement with what emerged from previous reports^56^. On the contrary, Bi_2_Te_2.2_Se_0.8_ exhibits a visible decrease in the scattering intensity with flake thickness d at ω_0_ = 66.7 cm^−1^ and ω_0_ = 76.7 cm^−1^, which becomes less pronounced at ω_0_ = 89.7 cm^−1^, such that, in all cases, the flakes become barely distinguishable (η_3_ ~ 1) from the substrate when d < 20 nm (Fig. 3d). The thickness and frequency dependences of s_3_ from Bi_2_Te_2.2_Se_0.8_ are consistent with a recent model^30^ of the near-field response formulated for Bi_2_Se_3_ which predicts deeply sub-diffractional, highly directional hyperbolic phonon-polaritons interacting with the electrons of the TSS. In particular, the experimental frequency dependence of Bi_2_Te_2.2_Se_0.8_ is retrieved if one includes a red-shift of the spectral features predicted by the model for Bi_2_Se_3_ towards the optical phonon frequencies of Bi_2_Te_3_ to describe Bi_2_Te_2.2_Se_0.8_, whose phonon frequencies reasonably approaches the ones of Bi_2_Te_3_^46^. A detailed simulation of the near-field response s_3_ of Bi_2_Te_3,_ as a function of the pumping frequency and of the flake thickness, is reported in the Supplementary Information (Supplementary Note 4 and Supplementary Fig. 15a). Consistently, in the Bi_2_Te_2.2_Se_0.8_ flakes (Fig. 3d), s_3_(ω_0_) is almost frequency independent in the 66.7 cm^−1^ < ω_0_ < 76.7 cm^−1^ range and then increases when ω_0_ = 89.7 cm^−1^, as we approach the peak of s_3_ due to the TO phonon at ω_to_^//^ = 95 cm^−1^$.$

A strong and sharp peak in near-field contrast is predicted at ω_to_^⊥^ ^30^, that in a Bi_2_Te_2.2_Se_0.8_ falls outside of the investigated spectral range (ω_to_^⊥^ = 50 cm^−1^ for the Bi_2_Te_3_)^46,47^. However, differently from the peak at ω_to_^//^, this peak is related to the far-field factor rather than to the near-field tip-sample interaction.

### Propagating collective excitations

**Fig. 4:**
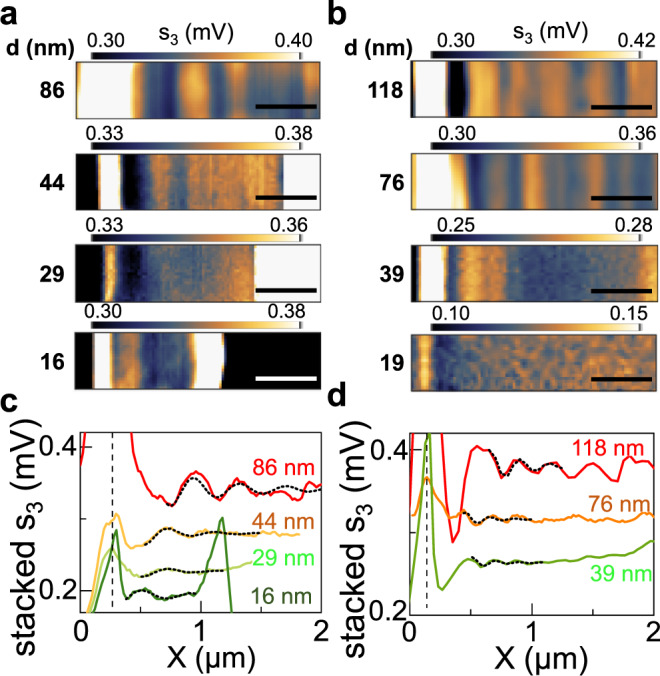
Real-space mapping of propagating excitations. **a**, **b** Near-field scattering maps of s_3_ acquired at 66.7 cm^−1^ at the edge of Bi_2_Se_3_ (**a**) and Bi_2_Te_2.2_Se_0.8_ (**b**) flakes, with different thicknesses d, indicated on the side. The scale bar corresponds to 500 nm. **c**, **d** Line profiles (colored solid lines) extracted averaging the maps in panels **a**, **b** vertically on the displayed window, showing signal oscillations as a function of the position X on flakes of Bi_2_Se_3_ (**c**) and Bi_2_Te_2.2_Se_0.8_ (**d**). The line profile corresponding to the map of the 19-nm-thick flake of Bi_2_Te_2.2_Se_0.8_ is not reported because it lacks evident periodicity. The line profiles are aligned to have the highest peak at the same position, which is identified by the black dashed vertical line. Fits of the oscillations to extract the periodicity, performed with the function described in the text, are plotted as short-dot black lines.

Figure 4 shows the near-field maps (Fig. 4a, b) of the edges of the Bi_2_Se_3_ and Bi_2_Te_2.2_Se_0.8_ flakes, acquired at ω_0_ = 66.7 cm^−1^ for a fixed cavity length L, and the related line profiles extracted by averaging over the displayed window along the vertical axis (Fig. 4c, d).

Analogous line profiles measured at ω_0_ = 76.7 and 90 cm^−1^ are reported in Supplementary Figs. 10, 11.

The near-field scattering maps (Fig. 4a, b) show a visible signal enhancement at the flake edges with the brightest edge corresponding to the side from which the THz beam enters in the s-SNOM.

A strong signal enhancement at the flakes edges has been previously identified in hBN as a signature of the activation of localized phonon-polariton modes^57^. The peaks localized at the flake edges have distinct thickness dependence in the two materials: in Bi_2_Se_3_ the peak broadens and increases its intensity with the thickness (Fig. 4c), while it appears always sharper, and with a less pronounced dependence of intensity on thickness in the Bi_2_Te_2.2_Se_0.8_ (Fig. 4d). We ascribe those peaks to edge resonances of phonon-polariton modes^57^, whose interplay with the main photoexcited modes quickly damps them close to the edge. Indeed, while the peaks retrieved in Fig. 4c, d are visibly edge modes, we observe signal intensity modulations in regions on flat topography (Fig. 4c, d), characterized by a much weaker amplitude, which we attribute to the interference of propagating modes launched by the tip and reflected at the flake edges. In our case, these oscillations in Bi_2_Se_3_ have comparable amplitude changing the thickness, while in Bi_2_Te_2.2_Se_0.8_ they display a less pronounced intensity for thinner flakes, becoming not distinguishable in the thinner flake (d = 19 nm).

To unveil the nature of the aforementioned propagating photoexcited modes, we analyze these signal oscillations with the function: ${s}_{3}(x)={{{\rm{Re}}}}\left(\frac{A}{\sqrt{x-{x}_{0}}}{{{{\rm{e}}}}}^{2{{{\rm{i}}}}{{{{\rm{q}}}}}_{{{{\rm{p}}}}}(x-{x}_{o})}\right)+B$, expected for modes launched by the tip and reflected by the flake edges^58^. In the fitting function, A is a complex-valued amplitude, B is a constant background, x_0_ represents the position of the reflecting interface taken as the flakes’ edge (dashed line in Fig. 4c, d), $1/\sqrt{x-{x}_{0}}$ is the geometrical decay and the exponential describes oscillations with periodicity $\frac{{{\rm{\pi }}}}{{q}_{p}}\,$, where the wavevector q_p_ is complex-valued q_p_ = q_1_ + iq_2_. The double period λ_p_ = $\frac{2{{{\rm{\pi }}}}}{{{{{\rm{q}}}}}_{1}}\,$ represents the wavelength of the photoexcited modes, whose frequency is set by the energy conservation law to match that of the impinging photon^59^.

**Fig. 5:**
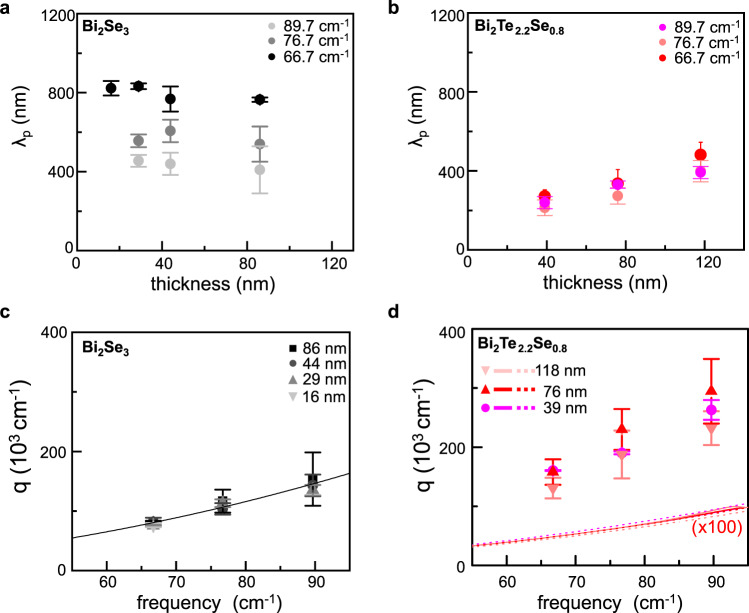
Energy dispersions of propagating modes. **a** Plasmon wavelength λ_p_ in Bi_2_Se_3_ as a function of thickness, extracted from the interference pattern measured at 66.7 cm^−1^ (black dots), at 76.7 cm^−1^ (gray dots), and at 89.7 cm^−1^ (light gray dots). **b** Wavelength λ_p_ of the photoexcited modes in Bi_2_Te_2.2_Se_0.8_ as a function of flake thickness extracted from the interference pattern measured at 66.7 cm^−1^ (red dots), at 76.7 cm^−1^ (light red dots), and 89.7 cm^−1^ (magenta dots). **c** Plasmon energy dispersion in Bi_2_Se_3_ for different flake thicknesses d ranging from 86 to 16 nm (colored dots) together with the predicted dispersion law (solid line). **d** Energy dispersion of the photoexcited modes in Bi_2_Te_2.2_Se_0.8_ for different flake thicknesses d ranging from 118 to 39 nm (colored dots), compared to the energy dispersion simulated for Dirac plasmons (colored solid lines) and massive bulk plasmons (colored dot lines) reported in Supplementary Fig. 12 and therein-discussed in detail. Error bars indicate the 95% confidence bands from the fit of interference patterns.

Figure 5a shows the obtained λ_p_ as a function of the flake thickness for Bi_2_Se_3_ and Bi_2_Te_2.2_Se_0.8_, respectively, at the three probed frequencies. In Bi_2_Se_3_, λ_p_ is almost thickness independent (Fig. 5a) at all probing frequencies. Vice versa in Bi_2_Te_2.2_Se_0.8_, there is a visible dependence of the oscillation period on d (Fig. 5b): λ_p_ decreases while reducing d.

The thickness-independent trend, extrapolated in Bi_2_Se_3,_ is compatible with photoexcited massive plasmons formed by the bulk charge carriers at the flakes surface, which give rise to a 2DEG, as a consequence of the band bending at the Bi_2_Se_3_ interface with adjacent materials^36^.

Conversely, the extrapolated thickness dependence of λ_p_ in Bi_2_Te_2.2_Se_0.8_ is expected either in presence of massive plasmons related to bulk charge carriers or for Dirac plasmons related to TSS^56,60^. It is worth mentioning that, in the limit of small wavevectors (qd < 1), Dirac plasmons dispersion can be approximated to the thickness-independent first-order dispersion^8^, while at larger wavevectors both the dependence on the thickness and on the bulk dielectric function emerge^60,61^.

We then investigate the experimental mode dispersion (Fig. 5c, d), for both material systems, and compare it with the theoretical trends predicted under the hypothesis discussed above. A quadratic dependence of the real part of the wavevector q_1_ = 2π/λ_p_ on the frequency ${{\upomega }}$_p_=${{\upomega }}$_0_ is observed for both materials. In Bi_2_Se_3_ the dispersion curve, calculated for massive plasmons formed by the 2DEG charge carriers^36^, perfectly matches our experimental data (Fig. 5c), confirming our claim. The dispersion of massive plasmons^30^ is evaluated using the relation ${{{\omega }}}_{{{{\rm{p}}}}}^{2}=\frac{{{{{\rm{e}}}}}^{2}}{2{\varepsilon }_{0}{\varepsilon }_{r}}\frac{{{{n}}}_{{{{\rm{M}}}}}}{{{m}}_{\rm {eff}}}{q}_{1}$, where ε_r_~6.3 is the average permittivity of the materials surrounding the flake (silica/silicon substrate and air), e is the electron charge, ε_0_ is the vacuum permittivity, n_M_ is the carrier density associated with the 2DEG and m_eff_  = 0.15 m_0_ is the effective mass^34^ of Bi_2_Se_3_, with m_0_ electron mass. Good agreement with the data is obtained considering a surface carrier density n_M_ = 3 × 10^10^ cm^−2^, which indicates charge-carrier depletion at the surface, in agreement with previous studies^62,63^ on Bi_2_Se_3_.

The theoretical dispersion curves predicted in Bi_2_Te_2.2_Se_0.8_ for massive bulk plasmons and for TSS Dirac plasmons are shown in Supplementary Fig. 12a, b, respectively. In the case of massive bulk plasmons, we use the same dispersion relation as for Bi_2_Se_3_ massive plasmon but with the effective mass^64^ of Bi_2_Te_3_ m_eff_ = 0.044 m_0_, and a thickness-dependent density for the bulk massive electrons n_M_(d). The thickness dependence is here encoded into the carrier density term n_M_ = n_s_ + C· n_bv_d^0.5^, which depends on the sheet carrier density n_s_, on the bulk carrier density n_bv_, and for which we consider a square-root dependence on the thickness d as in ref. ^65^. A bulk carrier density n_bv_ = 3 × 10^18^ cm^−3^ in Bi_2_Te_2.2_Se_0.8_ is taken from the value experimentally retrieved with direct Hall measurement in Bi_2_Te_2.4_Se_0.6_^66^, the coefficient C = 0.8 × 10^−5^ m^0.5^ is taken from ref. ^65^ and estimation of n_s_ = 2 × 10^12^ cm^−2^ is obtained by multiplying n_bv_ from ref. ^66^ for the thickness of the flake used for the n_bv_ determination (6 QL~8 nm). On the contrary, the dispersion of Dirac plasmons associated with TSS^56,60^ is: ${{{\omega }}}_{{{{\rm{p}}}}}^{2}=\frac{{{{{\rm{e}}}}}^{2}}{{\varepsilon }_{0}}\frac{{v}_{F}\sqrt{2{{{\rm{\pi }}}}{n}_{D}}}{h}\frac{{q}_{1}}{{{{{\rm{\varepsilon }}}}}_{{{{\rm{r}}}}}+{qd}{{{{\rm{\varepsilon }}}}}_{{{{\rm{TI}}}}}}$ where n_D_ is the Dirac carrier density (assumed equal to n_s_ to calculate the dispersion), *h* is the Planck constant, and ε_TI_(ω) is the Bi_2_Te_3_ permittivity^56^. The frequency dependence of ε_TI_ is crucial to account for the thickness dependence of the Dirac plasma frequency^67^, while here we use an average of the dielectric constants along the basal plane ε^⊥^ and along the c-axis ε^//^, computed as:$\,{{{{\rm{\varepsilon }}}}}_{{{{\rm{TI}}}}}=\sqrt{{\varepsilon }^{//}\cdot {\varepsilon }^{\perp }}\,$. Our model for the dielectric permittivity of Bi_2_Se_3_ and Bi_2_Te_3_ is detailed in Supplementary Note 4. The data of Supplementary Fig. 12a, b indeed clearly show that for Bi_2_Te_3_ none of these trends matches with our experimental data (Fig. 5d), and that, for both assumptions, we would expect much lower *q* values. This reflects in propagating plasmon wavelengths λ_p_ (Supplementary Fig. 12c, d) significantly longer (>100 μm), impossible to be captured via near-field nanoscopy.

We cannot, therefore, ascribe the oscillations observed in Bi_2_Te_2.2_Se_0.8_ to propagating bulk plasmons or Dirac plasmons. To shed further light on the nature of these modes we then perform numerical simulations of the collective mode energy dispersion in our Bi_2_Te_2.2_Se_0.8_ flakes, following the procedure described in ref. ^30^. We look for the dispersion of phonon-polariton modes in the presence of charge carriers populating the TSS, described by the surface sheet conductivity of Dirac fermions^68^, assuming that the chemical potential is located in the bulk bandgap, resulting in a negligible bulk conductance. The charge carriers in the TSS are expected to form Dirac plasmons that hybridize with the phonon-polariton modes to form hyperbolic plasmon–phonon-polaritons.

The near-field signal can be considered as a weighted average of the surface reflectivity for p- (or TM-) polarized light r_P_(q, ω_0_) over q. The dispersion of collective modes is identified by the maxima, at real arguments q and ω, of the imaginary part of the r_P_(q, ${{\omega }}$), ${{{\rm{Im}}}}\{{{\rm {r}}_{\rm {P}}}({\rm {q, ω}})\}$, which we calculate including the strong anisotropy of the frequency-dependent dielectric permittivity of Bi_2_Te_2.2_Se_0.8_ (see Methods).

**Fig. 6:**
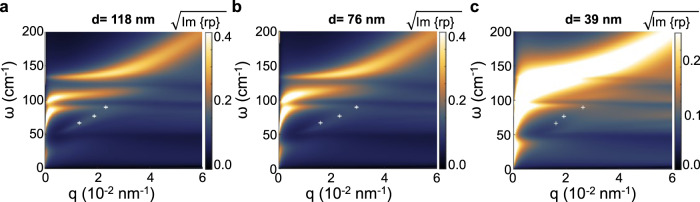
Collective mode dispersions. **a**–**c** Collective mode dispersions of Bi_2_Te_3_ slabs of different thickness d, rendered using the false color maps of Im{r_p_}. The parameters of the calculation are μ = 0.2 eV and **a** d = 118 nm; **b** d = 76 nm; **c** d = 39 nm. In the plots, the Fermi velocity is v_F_ = 0.5 × 10^8^ cm/s as for Bi_2_Te_3_ from ref. ^48^, the electron scattering rate is γ_e_ = 1 THz as in ref. ^4^, the vacuum and substrate permittivity are ε_0_ = 1, and ε_sub_ = 6.3. The white symbols correspond to the experimental data of Fig. 5d.

The false color maps of the function Im{r_P_(q, ω)} in Fig. 6 provide a convenient visualization of the collective mode spectra.

The bright lines in Fig. 6a–c are the dispersion curves of the collective modes calculated for Bi_2_Te_2.2_Se_0.8_ flakes of different thickness, assuming a constant chemical potential μ = 200 meV, which is chosen to fall within the electronic bandgap of the material (~280 meV^35^). The Im{r_p_} map calculated for chemical potentials μ = 50 meV, μ = 150 meV, and μ = 250 meV and for flake thickness 118 nm is reported in the Supplementary Fig. 14. In bismuth chalcogenide compounds, the electrochemical potential is indeed expected to approach the bottom of the conduction band as a consequence of the doping due to vacancies or antisite defects^69^. The smearing of the dispersion lines gives an idea of how damped the modes are.

The comparison with our experimental data (white crosses in Fig. 6a–c), show that our photoexcited modes all falls in the upper half of the hyperbolic band ω_to_^⊥^ < ω < ω_to_^//^ and their q values correspond to those expected for hybrid modes resulting from hybridization of hyperbolic phonon-polaritons with TSS Dirac plasmons into combined hyperbolic plasmon–phonon-polaritons modes (HP^3^ modes), and differ significantly from those expected when only Dirac plasmons come into play (see Supplementary Fig. 12b). The effect of the hybridization is encoded in the frequency shift of the dispersion curves when there is finite doping (see Supplementary Fig. 14). The damping of these modes is a consequence of the strong electron interaction with the charge carriers and increases with doping. From Fig. 6 we can also observe that the hyperbolic phonon-polariton modes fade away while decreasing the flake thickness. It is worth mentioning that the carrier density plays a fundamental role in dictating the observable activated excitations in our geometry, i.e., with wavelengths within the physical size of the exfoliated flakes. Indeed, in the case of Bi_2_Se_3_, the expected lower surface carrier density, combined with the larger effective mass m_eff_, entails the activation of short wavelength λ_p_ (submicron) massive plasmons, whereas these are not observable in Bi_2_Te_2.2_Se_0.8_ because the higher carrier density and lower m_eff_ push λ_p_ in the 0.5–1 mm range. Therefore, the ability to controllably tune the carrier density, e.g., by electrostatic gating, could open the possibility to selectively ignite the different phenomena.

Analogous activation of mixed plasmon and hyperbolic phonon-polaritons has been predicted for hBN thin slabs^30^, with the difference that the hyperbolic bands of hBN fall in the mid-infrared range, at much higher frequencies (around 1500 cm^−1^). Technological applications based on mid-infrared hyperbolic phonon-polaritons demonstrated for hBN, such as hyperlensing^70^, waveguiding^30^, and light focusing^71^, can be translated to the THz frequency range using Bi_2_Te_2.2_Se_0.8_ flakes. The hybridization of phonon-polaritons with Dirac plasmons introduces additional control knobs, namely flake thickness and doping, to tune the HP^3^ mode dispersion and accordingly the THz response of Bi_2_Te_2.2_Se_0.8_ flakes. Moreover, since HP^3^ modes are highly directional^30^, a change in the doping can be exploited to vary the propagation direction of the HP^3^.

In conclusion, we have provided the first direct experimental evidence of hyperbolic plasmon–phonon-polaritons modes at THz frequencies, and of the activation of massive bulk plasmons associated to band bending induced 2DEG, providing novel insights on the rich physics of TI materials. The interaction of the probed phonon-polaritons modes with the electrons from the TSS suggests possible ultrafast optical control^16^ by above bandgap photoexcitation to ideally switch on and off the hybridized modes, opening intriguing technological perspectives in plasmonics^72^, ultrafast photonics, spintronics^10^, nanophotonics^73,74^, and quantum optics^75^.

## Methods

### Growth

Single-crystalline ingots of Bi_2_Se_3_ and Bi_2_Te_2.2_Se_0.8_ were grown from melt by the vertical vacuum/inert atmosphere optimized Bridgman−Stockbarger method, following methods described in ref. ^76^. The resulting samples show extremely high chemical stability and do not manifest any effect related to degradation under air exposure.

### Total reflectivity of Bi_2_Te_2.2_Se_0.8_

The sample system is modeled as a stack of three materials: the air superstrate (labeled as 0) with permittivity ${{{{\rm{\varepsilon }}}}}_{0}$ = 1, from which the incident THz beam comes, the Bi_2_Te_2.2_Se_0.8_ flake of thickness d (labeled as 1) with sheet conductivity, ${{{\sigma }}}_{{{{\rm{SS}}}}}({{{\rm{q}}}},{{\omega }})$and the undoped silicon/silica substrate (labeled as 2) with permittivity ε_s_ = 6.3. Following ref. ^30^, the P-polarization reflectivity r_P_ of this layered system is given by:

1 $${{{{\rm{r}}}}}_{{{{\rm{P}}}}}=\frac{{r}_{12}\left({r}_{01}+{r}_{10}-1\right)-{r}_{01}{e}^{(-2i{k}_{//}d)}}{{r}_{10}{r}_{12}-{e}^{(-2i{k}_{//}d)}}$$

where k_//_ = $\sqrt{{\varepsilon }^{\perp }}\sqrt{\frac{{\omega }^{2}}{{c}^{2}}-\frac{{q}^{2}}{{\varepsilon }^{//}}}$ is the out-of-plane momentum inside the flake, and ε^⊥^ and ε^//^ are the in-plane and out-of-plane dielectric permittivities calculated from the parameters available in the literature for Bi_2_Te_3_ as detailed in the supplementary information. The term r_12_ is the reflectivity at the flake/substrate interface and r_01_, r_10_ are the reflectivities at the air/flake interface as coming from the air and from the flake side, respectively. These latter are computed as follows in cgs units:

$${r}_{01}=\frac{{\varepsilon }^{\perp }{k}_{0}-{\varepsilon }_{0}{k}_{//}+\frac{4\pi }{\omega }{\sigma }_{{SS}}{k}_{0}{k}_{//}}{{\varepsilon }^{\perp }{k}_{0}{+\varepsilon }_{0}{k}_{//}+\frac{4\pi }{\omega }{\sigma }_{{SS}}{k}_{0}{k}_{//}}{{{\rm{;}}}}$$

$${r}_{10}=\frac{{\varepsilon }_{0}{k}_{//}-{\varepsilon }^{\perp }{k}_{0}+\frac{4\pi }{\omega }{\sigma }_{{SS}}{k}_{//}{k}_{0}}{{{\varepsilon }_{0}{k}_{//}+\varepsilon }^{\perp }{k}_{0}+\frac{4\pi }{\omega }{\sigma }_{{SS}}{k}_{//}{k}_{0}}{{{\rm{;}}}}$$

2 $${r}_{12}=\frac{{{{{\rm{\varepsilon }}}}}_{{{{\rm{s}}}}}{k}_{//}-{\varepsilon }^{\perp }{{{{\rm{k}}}}}_{{{{\rm{s}}}}}+\frac{4\pi }{\omega }{{{{\rm{\sigma }}}}}_{{{{\rm{SS}}}}}{{{{\rm{k}}}}}_{{{{\rm{s}}}}}{k}_{//}}{{{{{{\rm{\varepsilon }}}}}_{{{{\rm{s}}}}}{k}_{//}+\varepsilon }^{\perp }{{{{\rm{k}}}}}_{{{{\rm{s}}}}}+\frac{4\pi }{\omega }{{{{\rm{\sigma }}}}}_{{{{\rm{SS}}}}}{{{{\rm{k}}}}}_{{{{\rm{s}}}}}{k}_{//}}$$

${{{{\rm{k}}}}}_{{{{\rm{s}}}}}$ and ${{{{\rm{k}}}}}_{0}$ are the out-of-plane momenta at the substrate and in the air and are defined in analogy to that in the flake but considering that they are isotropic materials: ${{{{\rm{k}}}}}_{{{{\rm{s}}}}}=\sqrt{{{{{\rm{\varepsilon }}}}}_{{{{\rm{s}}}}}}\sqrt{\frac{{\omega }^{2}}{{c}^{2}}-\frac{{q}^{2}}{{{{{\rm{\varepsilon }}}}}_{{{{\rm{s}}}}}}}$ and ${{{{\rm{k}}}}}_{0}=\sqrt{\frac{{\omega }^{2}}{{c}^{2}}-{q}^{2}}$. Finally, the sheet conductivity ${{{{\rm{\sigma }}}}}_{{{{\rm{SS}}}}}$ is evaluated as:

$${{{{\rm{\sigma }}}}}_{{{{\rm{SS}}}}}=\frac{{{{\rm{i}}}}\omega }{{q}^{2}}{{{{\rm{e}}}}}^{2}P$$

with P polarizability of Dirac fermions^30,68^, in which the Fermi velocity of the material v_F_ = 0.5 × 10^8^ cm/s and the chemical potential μ enter.

By using the same ${{{{\rm{\sigma }}}}}_{{{{\rm{SS}}}}}$ in r_01_ and in r_12_ we are assuming the same sheet conductivity at both the interfaces air/flake and flake/substrate. The sheet conductivity is nonzero only for finite doping and determines the appearance of the Dirac plasmon modes and their hybridization with hyperbolic phonon-polaritons to form HP^3^ excitations.

## Supplementary information

Supplementary Information

## Data Availability

The data that support the plots within this paper and other findings of this study are available from the corresponding authors upon reasonable request.

## Code availability

The codes and simulation files that support the plots and data analysis within this paper are available from the corresponding author upon reasonable request.
