# Publisher Correction: Mapping propagation of collective modes in Bi_2_Se_3_ and Bi_2_Te_2.2_Se_0.8_ topological insulators by nearfield terahertz nanoscopy

**DOI:** 10.1038/s41467-021-27558-0

**Published:** 2021-12-14

**Authors:** Eva Arianna Aurelia Pogna, Leonardo Viti, Antonio Politano, Massimo Brambilla, Gaetano Scamarcio, Miriam Serena Vitiello

**Affiliations:** 1 NEST, CNR-Istituto Nanoscienze and Scuola Normale Superiore, Piazza San Silvestro 12, 56127 Pisa, Italy; 2 Department of Physical and Chemical Sciences, University of L’Aquila, via Vetoio 1, 67100 L’Aquila, Italy; 3 Dipartimento Interateneo di Fisica, Università degli Studi e Politecnico di Bari, and CNR-Istituto di Fotonica e Nanotecnologie, via Amendola 173, 70126 Bari, Italy

**Keywords:** Topological insulators, Electronic properties and materials

Correction to: *Nature Communications* 10.1038/s41467-021-26831-6, published online 18 November 2021.

The original version of this Article had a few typesetting errors in the main text. In the section “Results and discussion”, subsection “Propagating collective excitations”, the 4th paragraph, the texts “a less pronounced dependence of intensity on thickness” were mistakenly written as “a less pronounced thickness-dependent intensity”. In the same subsection, the 9th paragraph, the equation “${\omega }_{{{{{{\rm{p}}}}}}}^{2}=\frac{{{{{{{\rm{e}}}}}}}^{2}}{2{\varepsilon }_{0}{\varepsilon }_{{{{{{\rm{r}}}}}}}}\frac{{n}_{{{{{{\rm{M}}}}}}}}{{m}_{{{{{{\rm{eff}}}}}}}}{q}_{1}$” was mistakenly written as “${\omega }_{{{{{{\rm{p}}}}}}}^{2}=\frac{{{{{{{\rm{e}}}}}}}^{2}}{4{\varepsilon }_{0}{\varepsilon }_{{{{{{\rm{r}}}}}}}}\frac{{n}_{{{{{{\rm{M}}}}}}}}{{m}_{{{{{{\rm{eff}}}}}}}}{q}_{1}$” and the equation “$n_{M}=3 \times10^{10} {\rm cm}^{-2}$” was mistakenly written as “$n_{M}=6 \times10^{10} {\rm cm}^{-2}$”. These errors have been corrected in both the PDF and HTML versions of the Article.

The original version of this Article also had errors in one figure of the Supplementary Information. In Supplementary Fig. 6, the “$\varepsilon_1$”, “$\varepsilon_2$” axis of panel b,c were mis-aligned. In the same figure, the “Frequency” axis corresponding to the “${{{\mathrm{Im}}}}\{\varepsilon_{{{\mathrm{bulk}}}}\}$” axis of panel e was mis-aligned. In the caption of Supplementary Figure 6c the text “(c) for d_1_= 0.1 and d_1_= 4.8 nm” was mistakenly written as “(c) for d_1_= 4.8 and d_1_= 0.1 nm”. These have all been corrected in the new Supplementary Information.

## Supplementary information

Updated Supplementary Information

