# Supplementary material for: Mapping propagation of collective modes in Bi2Se3 and Bi2Te2.2Se0.8 topological insulators by near-field terahertz nanoscopy
Source: Nat Commun. 2021 Nov 18;12:6672. doi: 10.1038/s41467-021-26831-6 (PMC8602307; doi:10.1038/s41467-021-26831-6)
Supplement: Supplementary file 1 — Supplementary Information [file 41467_2021_26831_MOESM1_ESM.pdf]

Supplementary Information

Mapping propagation of collective modes in Bi_2Se_3 and $Bi_2Te_{2.2}Se_{0.8}$ topological insulators by near-field terahertz nanoscopy

Eva Arianna Aurelia Pogna[1], Leonardo Viti[1], Antonio Politano[2], Massimo Brambilla[3], Gaetano Scamarcio,[3] Miriam Serena Vitiello[1]

[1]*NEST, CNR-Istituto Nanoscienze and Scuola Normale Superiore, Piazza San Silvestro 12, 56127 Pisa, Italy*
[2]*Department of Physical and Chemical Sciences, University of L'Aquila, via Vetoio 1, 67100 L'Aquila, Italy*
[3]*Dipartimento Interateneo di Fisica, Università degli Studi e Politecnico di Bari, via Amendola 173, 70126 Bari, Italy*

Supplementary Note 1: Micro-Raman spectroscopy

In order to evaluate the crystalline quality of the exfoliated topological insulator (TI) Bi_2Se_3 and $Bi_2Te_{2.2}Se_{0.8}$ flakes we perform micro-Raman spectroscopy. We excite the individual flakes with the frequency doubled line of a Nd:YAG pumping laser (532 nm) with an optical power density of 0.4 mW/μm^2, and with a spatial lateral resolution of about 2 μm. The Raman spectra show good crystalline quality for both materials. The micro-Raman spectrum of the Bi_2Se_3 (Supplementary Fig. 1a) includes the characteristics E_g^1, A_{1g}^1, E_g^2, A_{1g}^2 vibrational modes located at 36 cm^{-1}, 72 cm^{-1}, 130 cm^{-1} and 172 cm^{-1}, respectively, in agreement with previous measurements of thin Bi_2Se_3 [1]. The same peaks are also retrieved for $Bi_2Te_{2.2}Se_{0.8}$ (Supplementary Fig. 1b) and, except for the E_g^1, they are all red-shifted with respect to Bi_2Se_3. The frequency position of E_g^1, A_{1g}^1, E_g^2, A_{1g}^2 peaks is 36 cm^{-1}, 62 cm^{-1}, 101 cm^{-1} and 135 cm^{-1}, respectively, differing only by 1-3 cm^{-1} from what reported for thin films of Bi_2Te_3[2].

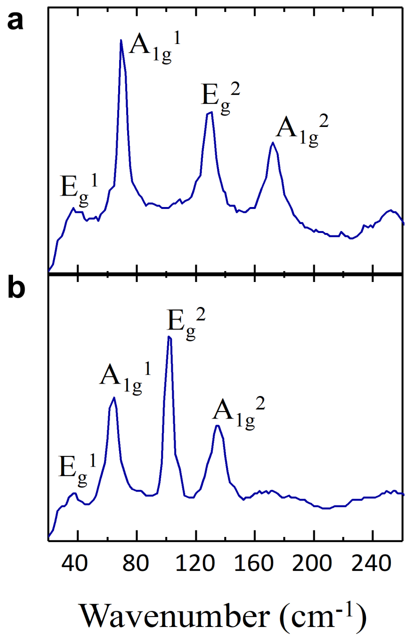

Supplementary Figure 1: Raman characterization. Micro-Raman spectra of a 40 nm thick Bi_2Se_3 flake (a) and a 30 nm thick $Bi_2Te_{2.2}Se_{0.8}$ flake (b) measured with a laser wavelength of 532 nm.

Supplementary Note 2: Time domain THz-nanoscopy

Time-domain THz-nanoscopy is performed coupling the TDS system to a commercial s-SNOM from Neaspec/attocube.

2.1 Fixed time delay maps

In Supplementary Fig. 2 we report the TDS-maps of two thick flakes (d > 70 nm) of Bi_2Se_3 and $Bi_2Te_{2.2}Se_{0.8}$ at a fixed time delay t corresponding to maximum field amplitude. The scattered field amplitude s_n is higher on the flake compared to the substrate and, by increasing the demodulation order n, the signal decays more rapidly with tip-to-sample distance such that background artifacts are reduced. The TDS spectra in Figure 1 of the main text are obtained by averaging time-delay scans acquired at five different positions on each of the two flakes in Supplementary Fig. 2.

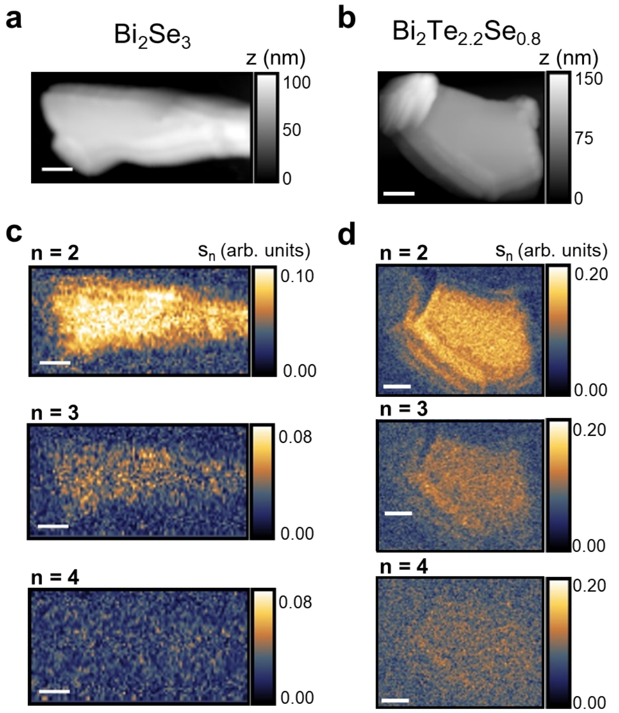

Supplementary Figure 2: Near-field hyperspectral imaging of topological insulators. a-b Topography of two flakes of Bi_2Se_3 (a) and $Bi_2Te_{2.2}Se_{0.8}$ (b). **c-d** Near-field TDS maps of Bi_2Se_3 (c) and $Bi_2Te_{2.2}Se_{0.8}$ (d) at a fixed time delay t and at different demodulation order n= 2, 3, 4 showing how the signal amplitude decreases with increasing n; scale bars correspond to 500 nm.

2.2 Spatial resolution and far-field background

In our nanoscopy experiments, the tip is operated in tapping mode with oscillation frequency Ω. The near-field signal depends non-linearly on the tip-sample distance as it is shown in Supplementary Fig. 3.

The near-field interaction decreases over distances comparable to the tip radius (tens of nanometers), whereas the background varies on the radiation wavelength scale (tens of microns for THz frequency light). Accordingly, for tapping amplitudes < 500 nm, the background slowly changes and it contributes mostly to the dc term and to the lower harmonics.

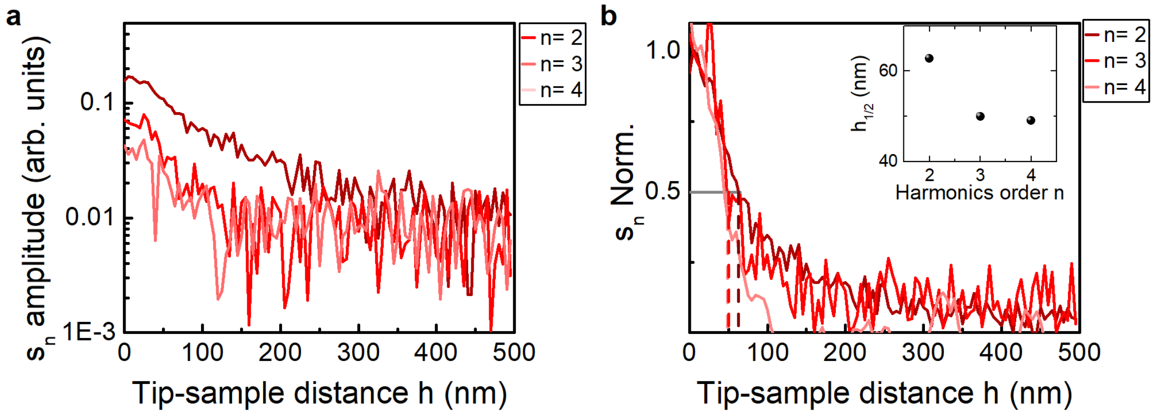

Supplementary Figure 3: Approach curves for hyperspectral nanoimaging measurements. a-b Approach curves at the harmonics of the tapping frequency of order n= 2, 3, 4 recorded with TDS system on gold for fixed time delay corresponding to an amplitude peak of the THz pulse, reported in semi-logarithmic scale (a); in linear scale (b) to calculate the distance $h_{1/2}$, at which the normalized signal drops to half of its maximum. Inset: $h_{1/2}$ plotted as a function of the order n of the harmonic.

To find a good compromise between signal intensity and suppression of the background due to non-local scatterers, we select the harmonic n= 2 to analyze the near-field maps, being the lowest order Fourier component at which the signal reaches the noise level once retracting the tip from the sample in Supplementary Fig. 3. At n= 2 and higher order harmonics, the signal reaches the same value for large tip-sample distance, corresponding to the noise level of our detection system. The decay distance $h_{1/2}$, at which the signal amplitude decays by a factor of 2, is equal to 63 nm at n= 2 and decreases with the demodulation order n, due to the virtual tip sharpening effect[3]. We then characterize the signal amplitude s_n and the lateral resolution Δx by analyzing the line profiles across the interface between an Au marker and the Si substrate at a fixed time delay (see Supplementary Fig. 4).

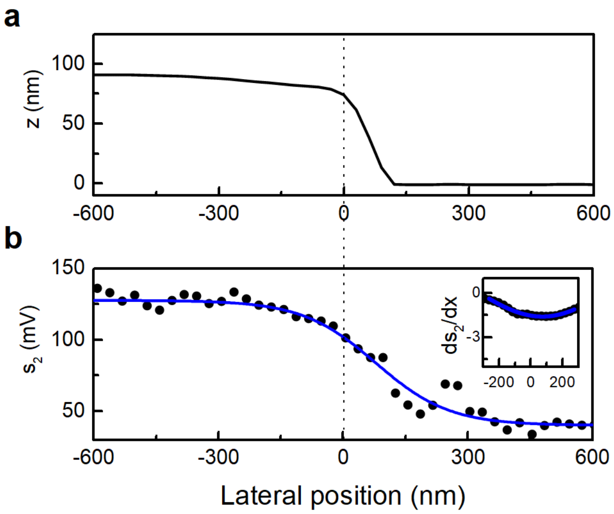

Supplementary Figure 4: Spatial resolution of hyperspectral nanoimaging measurements. **a** Topography scan of Au marker along a line orthogonal to its edge, taking the substrate as z = 0 reference. **b** THz near-field self-mixing signal at the second harmonic s_2 (black dots) from the hyperspectral nanoimaging experiment evaluated at a fixed time delay on the same line as panel (a) together with the best fit using sigmoidal function (solid blue line). The first derivative of the signal ds_2/dx (black dots) is reported in the inset, together with Lorentzian fit function (blue line) used to estimate the lateral spatial resolution Δx.

We define the spatial resolution, in analogy to other microscopy techniques, as the full width at half maximum (FWHM) of the line spread function (LSF). The LSF can be calculated by taking the derivative of the line profile measured across an interface. Supplementary Fig. 4 shows a line scan at the edge of a gold marker together with the its first-derivative which is fit with a Lorentzian function with FWHM of 260 ± 20 nm that can be considered as an estimation of our spatial resolution Δx.

Supplementary Note 3: Modeling of the near field response

3.1 Bulk model

Three dimensional model calculations of the near-field contrast η based on the well-established Finite dipole approximation[4–6], considering multiple longitudinal optical (LO) and transverse optical (TO) phonon modes, both in // and ⊥ directions, are reported in the Supplementary Fig.

5a. To simulate the contrast, we adopt a constant scattered near-field signal s_n from Au (as numerically retrieved from the available dispersion data) and we use the vibrational modes of Bi_2Te_3 to model the $Bi_2Te_{2.2}Se_{0.8}$ flake, due to the low dispersion of the phonon modes of the $Bi_2(Te_{(1-x)}Se_x)_3$ alloy at x <1/3. To account for the strong p-polarization of the electric field funneled by the tip[7], we consider only the p-wave contribution to the flake reflectivity given by the Fresnel formula[8] for uniaxial crystal cut perpendicularly to the c-axis. The model reproduces the experimental spectra, suggesting that Bi_2Te_3 can reasonably mimic the near-field response of the $Bi_2Te_{2.2}Se_{0.8}$. The same set of simulations also predicts that the interaction with a specific optical phonon is accompanied by a shift in the phase ϕ of the scattered field with a strongest modulation occurring at the frequency for which η_{Au} is minimum (see Supplementary Fig. 5b).

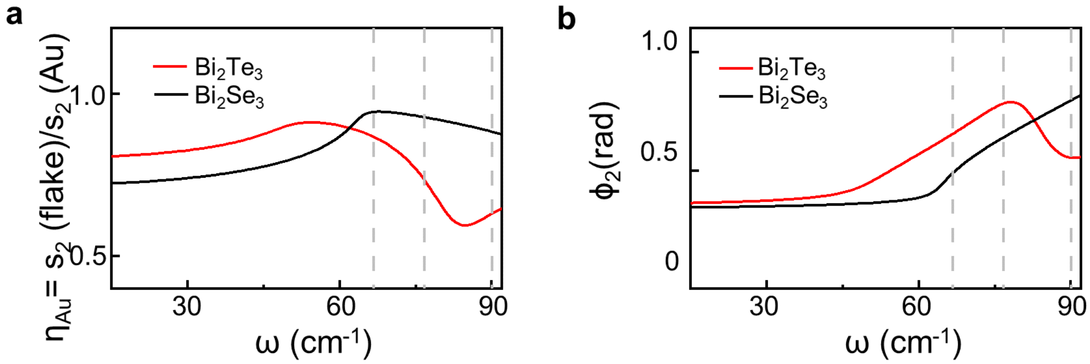

Supplementary Figure 5: Bulk phonons near-field response. a-b Simulated near-field contrast η_2 (a) and phase ϕ_2 (b) of second-order demodulated signal s_2 as a function of frequency for bulk Bi_2Se_3 (black line) and Bi_2Te_3 (red line).

3.2 Multilayer scattering model

In order to disentangle the dielectric response from the bulk and the surface, we combine the finite-dipole approach with a multilayer model and we implement an inversion

algorithm to extract the local dielectric function of the surface layer alone, Refs. 9,10. The multilayer structure (Supplementary Fig. 6a) includes four media: air, the surface states under study, the bulk TI, and the SiO_2/Si substrate.

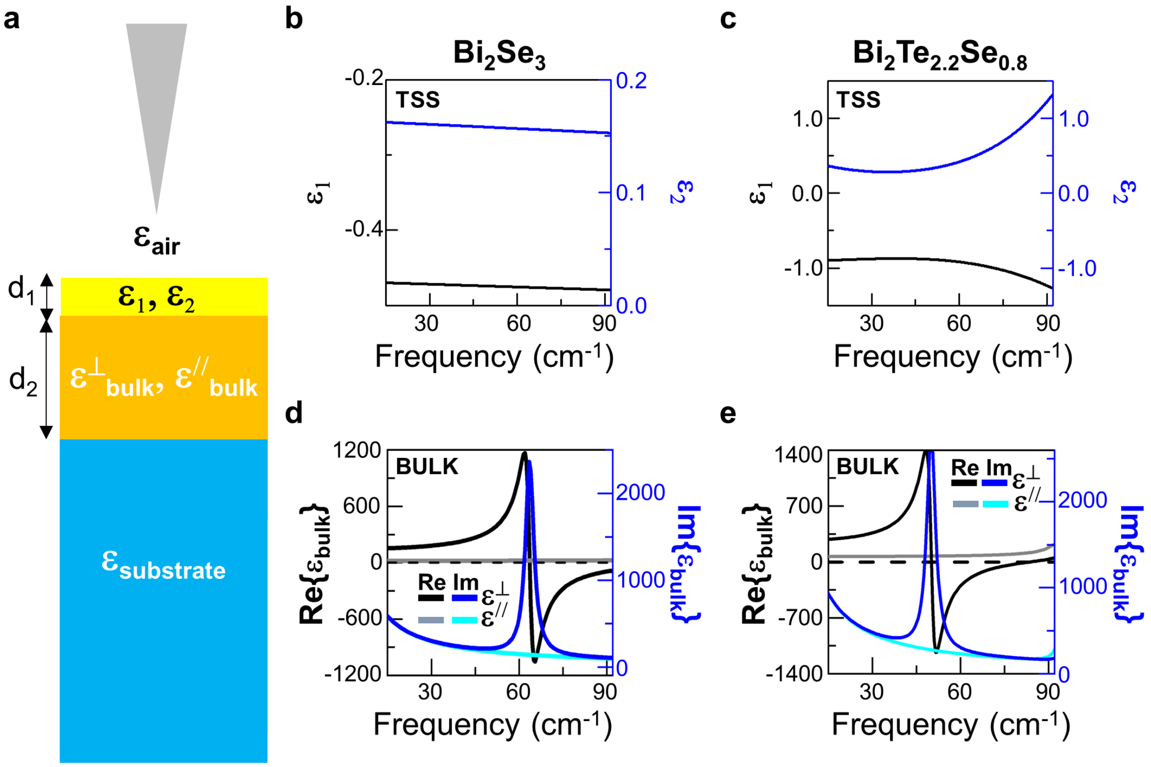

Supplementary Figure 6: Near-field tomography. a Sketch of the multilayer model with sample structure and the input parameters used in the retrieval algorithm to analyze the near-field spectra of Fig. 1b-c. **b-c** Real and imaginary part of the dielectric permittivity associated to the surface states extracted in Bi_2Se_3 (b) and $Bi_2Te_{2.2}Se_{0.8}$ (c) for d_1= 0.1 nm and d_1= 4.8nm, respectively. **d-e** Permittivity along the directions parallel (//) and perpendicular (⊥) to the c-axis that describes the bulk response of Bi_2Se_3 (d) and $Bi_2Te_{2.2}Se_{0.8}$ (e).

To extract the TSS permittivities from the inversion algorithm, we have to start from the knowledge of the thickness and the permittivity of all the other layers. The bulk TI is described as a layer of thickness d_2 with bulk dielectric permittivity which includes both the contribution of the bulk optical phonons (see Supplementary Note 4.5), which is anisotropic, and the contribution of a Drude gas of electrons with effective carrier density n= 3 × 10^{18} cm^{-3} Ref. 11. The electron scattering rates, γ_{Drude}= 190 THz (6338 cm^{-1}) for $Bi_2Te_{2.2}Se_{0.8}$ and γ_{Drude}= 85 THz (2835 cm^{-1}) for

Bi_2Se_3, are evaluated from the mobility μ_e measured on the same flakes,[12] using the relation: $\mu_e = e/(\gamma_{Drude} m_{eff})$, where m_{eff} is the electron effective mass of the material. For the SiO_2/Si substrate underneath, we use literature values for the dielectric functions (ε= 6.3) and ε= 1 for air. The surface states are finally described by a layer of thickness d_1 with unknown permittivity $\varepsilon_{TSS} = \varepsilon_1 + i\varepsilon_2$. In analogy to Ref. 9, we make no assumption on the frequency dependence of TSS, and we also leave the surface state thickness as an optimization parameter.

The near-field signal expected from this multilayer structure is calculated as the product $s=(1+r_p)^2 \alpha_{eff}(z,\omega)$, with r_p is the reflectivity of the sample for p-polarized light and α_{eff} is the tip-sample polarizability. We evaluate $\alpha_{eff}(z,\omega)$ as a function of frequency ω and tip-sample distance, i.e. the tip height z, within the framework of the finite dipole model using the following parameters to model the tip: permittivity $\sum_{tip}$= -100000+i215000, apex radius r= 40 nm, effective length L_1= 530 nm, fraction of the total charge induced on tip equal to g= 0.98 $e^{0.08i}$. The tapping motion of the tip is described by a sinusoidal variation of z and the near-field intensity s_n and phase ϕ_n at the demodulation order *n* are then obtained from the Fourier series over an oscillation cycle. By minimizing the deviation of the calculated signal from the experimentally measured contrast in Fig.1b to be within the experimental noise level, we extract the local dielectric function associated to the surface state. This leads to the calculated scattered amplitude and phase in Fig. 1d, 1e, which are then compared with the experimental data of Fig. 1b,1c.

Supplementary Figs. 6b-e show the frequency dependence of the real and imaginary part of the dielectric function of the surface state (Supplementary Fig. 6b-c) and that of the bulk (Supplementary Fig. 6d-e), as extrapolated from our experimental data for Bi_2Se_3 (Supplementary Fig. 6b, 6d) and $Bi_2Te_{2.2}Se_{0.8}$ (Supplementary Fig. 6c, 6e). The method well reproduces our experimental data, if we consider a surface layer thickness of 4.8nm for the $Bi_2Te_{2.2}Se_{0.8}$, in good agreement with the values retrieved in Ref. 9 while for Bi_2Se_3 the spectral response is uniquely

determined by the response of the bulk TI, since the model deviation from the data is minimized for $d_1 < 1$ nm.

Supplementary Note 4: Detectorless self-mixing interferometric nanoscopy.

Detectorless self-mixing interferometric nanoscopy is performed coupling our THz-QCL sources to a commercial s-SNOM from Neaspec/attocube.

4.1 Self-mixing interferometry near-field maps

Supplementary Figs. 7a-b show the near-field maps of the flakes described in the main text (A1-A4 for Bi_2Se_3 and B1-B4 for $Bi_2Te_{2.2}Se_{0.8}$), acquired at 66.7 cm^{-1}, 76.7 cm^{-1}, 89.7 cm^{-1} via the detectorless self-mixing interferometric nanoscopy.

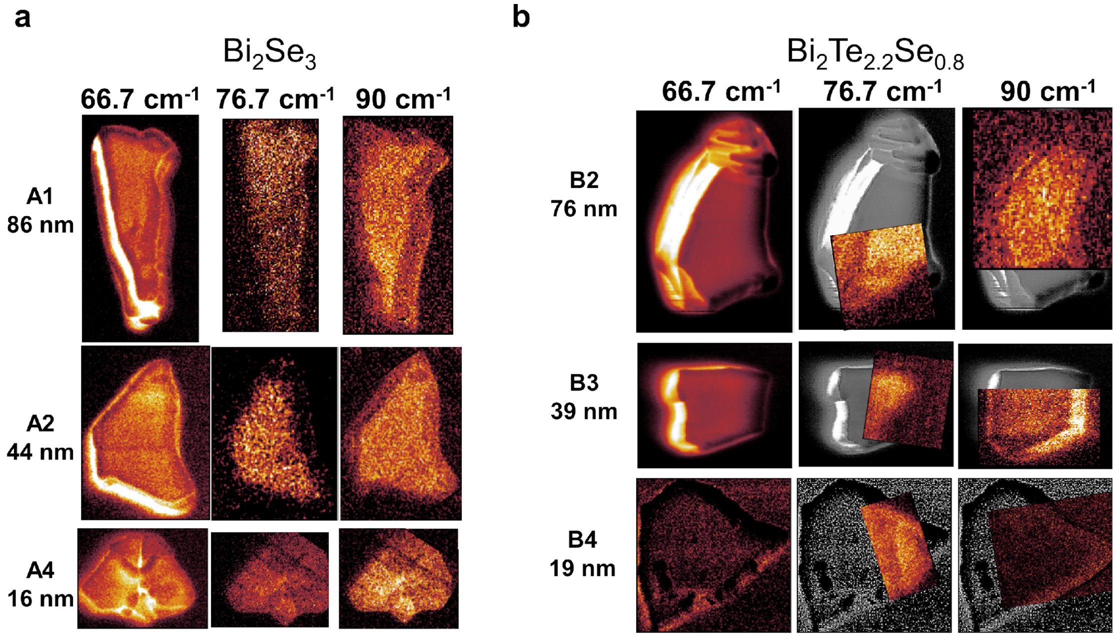

Supplementary Figure 7: Self-mixing interferometry near-field maps. **a-b** Comparison of the scattered near-field maps acquired on Bi_2Se_3 (a) and $Bi_2Te_{2.2}Se_{0.8}$ (b) flakes of different thickness, at frequency ω_0= 66.7, 76.7, 90 cm^{-1}, recorded by demodulating the self-mixing signal at the third harmonic of the tapping frequency. In panel b) the self-mixing maps are presented as overlapped to the AFM maps (gray scale) of the same flake to highlight the scan region.

The spatial resolution of the self-mixing interferometry experiment is evaluated through the analysis of the signal variation at the edge of a 50 nm high Au marker (Supplementary Fig. 8). The first derivative of the step-like line profile in Fig. S8b is then fitted with a Lorentzian function with FWHM of 30 ± 5 nm.

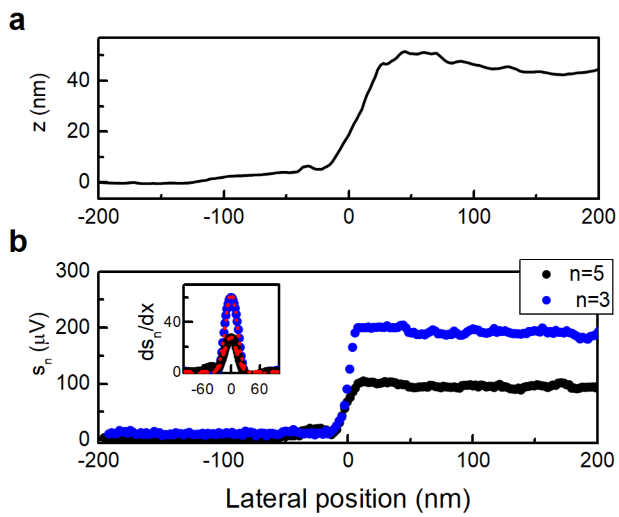

Supplementary Figure 8: Spatial resolution self-mixing interferometry near-field maps. **a** Topography line scan of an Au marker. **b** Near-field profiles of the self-mixing signal at the fifth (s_5) and third (s_3) demodulation order corresponding to the line-scan of panel (a) measured with THz QCL emitting at 76.7 cm^{-1}. The first derivatives of s_3 (blue dots) and s_5 (black dots) are reported in the inset together with the fits with Lorentzian functions (red dotted and dashed lines) used to determine the spatial resolution Δx as FWHM.

4.2 Self-mixing fringes analysis

Supplementary Figs 9a-f show the phase extracted from the analysis of the self-mixing fringes while moving along a line-scan across the flake/substrate boundary in a set of Bi_2Se_3 (Supplementary Figs. 9a-c) and $Bi_2Te_{2.2}Se_{0.8}$ flakes (Supplementary Fig. 9d-f). The position of the edge corresponds to x= 0, for x< 0 the tip is on the substrate and for x> 0 on the flake.

The phase jump is almost thickness independent in Bi_2Se_3, while it shows a visible dependence from the flake thickness in $Bi_2Te_{2.2}Se_{0.8}$ only at the highest pumping frequency (89.7 cm^{-1}). In all cases, although the retrieved trends do not perfectly match those expected (Fig. 1d, main text), the retrieved phase jumps are in reasonable agreement with the predicted values, for all thickness values.

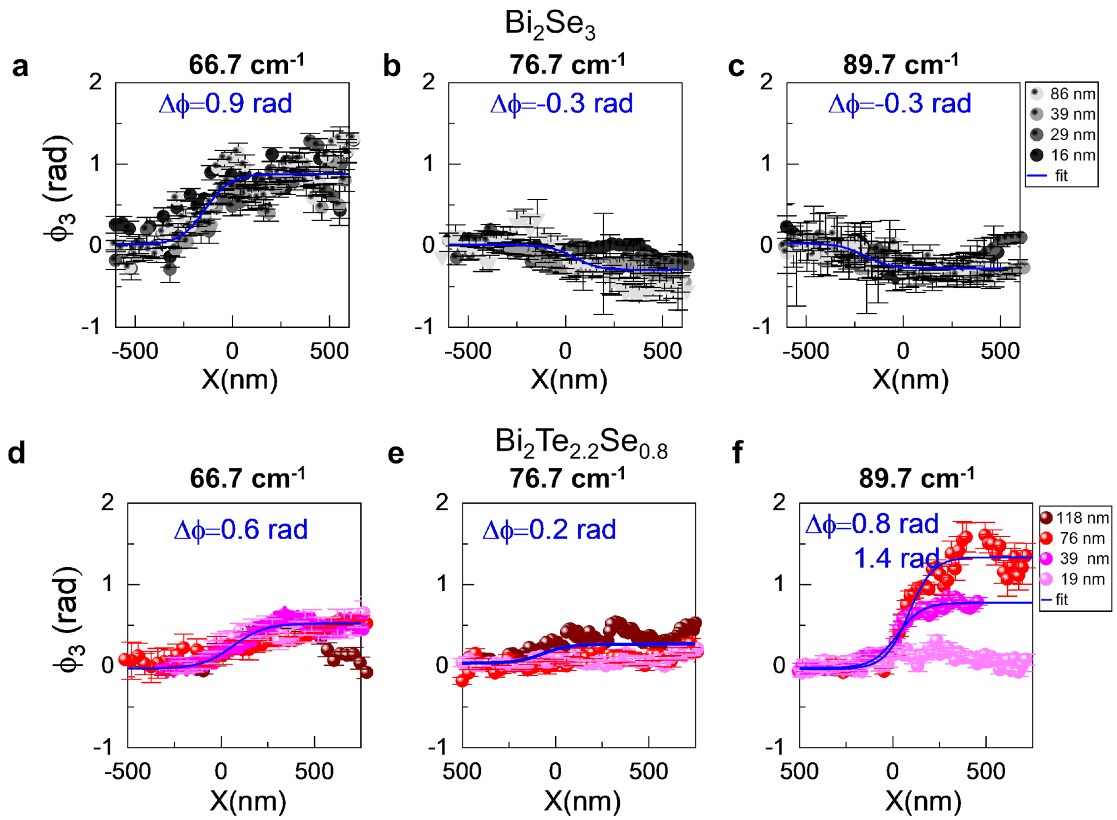

Supplementary Figure 9: Self-mixing fringes phase. **a-c** Phase ϕ_3 of the third-order near-field signal s_3 extracted from the fit of the self-mixing fringes while moving on the sample from the substrate to the flakes of Bi_2Se_3 at three different pumping frequencies 66.7 cm^{-1} (a), 76.7 cm^{-1} (b) and 89.7 cm^{-1} (c) for different flake thickness as indicated in the legend of panel (c). **d-f** Phase variation while moving from the substrate to the flakes of $Bi_2Te_{2.2}Se_{0.8}$ at three different pumping frequencies 66.7 cm^{-1} (d), 76.7 cm^{-1} (e) and 89.7 cm^{-1} (f), for different flake thicknesses as indicated in the legend of panel (f). For both materials, we use these trends to evaluate the phase variation $\Delta\phi_3$ between substrate and flake reported in Fig.3 of the main text. Error bars are the 95% confidence bands from the sinusoidal fit of the self-mixing fringes.

4.3 Signal at the flake edges

Supplementary Figures 10 and 11 show the n= 3 near-field signals collected close to the edge of flakes of Bi_2Se_3 (Supplementary Fig. 10) and $Bi_2Te_{2.2}Se_{0.8}$ (Supplementary Fig. 11) of different thickness, with QCL sources emitting at 76.7 cm^{-1} and 89.7 cm^{-1}.

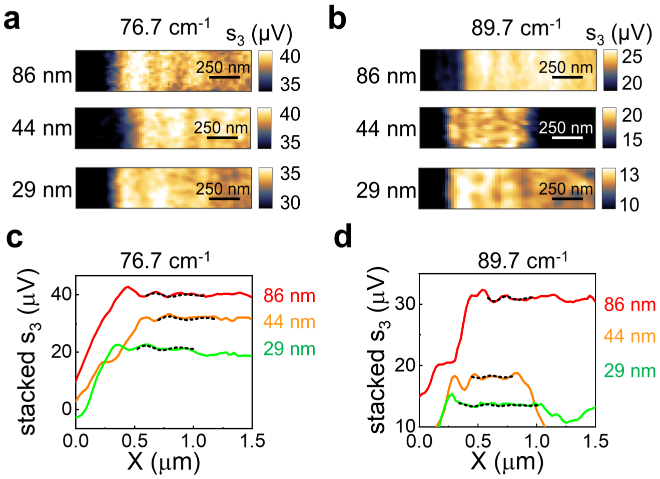

Supplementary Figure 10: Oscillations analysis in Bi_2Se_3. a-b Near-field scattering signal (s_3) maps at the edges of Bi_2Se_3 flakes of different thickness from 86 nm to 29 nm measured at 76.7 cm^{-1} (a) and 89.7 cm^{-1} (b). **c-d** Line profiles extracted from the maps in (a-b) averaging along the vertical direction (colored solid lines). The signal intensity oscillations observed by moving from the edge to the flake center (from left to right) in (c,d) are analyzed with the function described in the text to extract the periodicity λ_p. The best fit is reported as black short dotted lines.

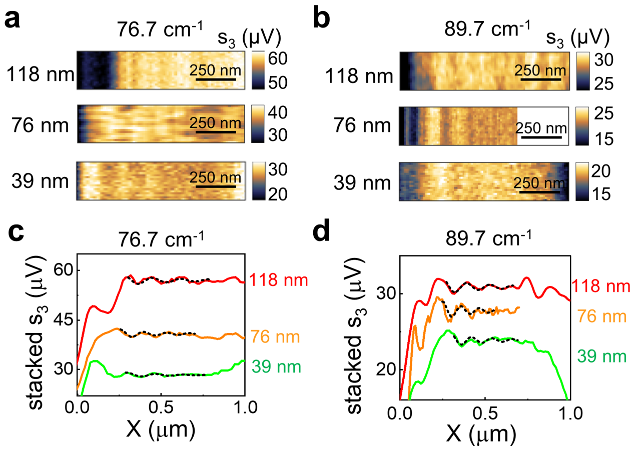

Supplementary Figure 11: Oscillations analysis in $Bi_2Te_{2.2}Se_{0.8}$. a-b Near-field scattering signal (s_3) maps at the edges of $Bi_2Te_{2.2}Se_{0.8}$ flakes of different thickness from 118 nm to 39 nm measured at 76.7 cm^{-1} (a) and 89.7 cm^{-1} (b). **c-d** Line profiles extracted from the maps in (a-b) averaging along the vertical direction (colored solid lines). The signal intensity oscillations observed by moving from the edge to the flake center (from left to right) in (c,d) are analyzed with the function described in the text to extract the periodicity λ_p. The best fit is reported as black short dotted lines.

The near-field self-mixing maps are analyzed to identify fingerprints of interference patterns due to the superposition of propagating polariton modes launched by the tip and reflected at the flake

edges. Line profiles are extracted from the maps along cuts with direction orthogonal to the edges. The signal oscillations are analyzed with the function $s_3(x) = \mathrm{Re}\left(\frac{A}{\sqrt{x-x_0}} e^{2iq_p(x-x_0)}\right) + B$ described in the main text to extract the periodicity λ_p shown in Figure 5, as a function of the flake thickness and of the laser frequency. To fit the data, we exclude the first highest peak at the flake edge, which can include contributions arising from the edge modes. The oscillations appear damped (only less than 3 periods can be observed) and they show higher amplitude for a signal demodulation order n= 3 than for higher orders.

4.4 Bulk and Dirac Plasmon dispersion in $Bi_2Te_{2.2}Se_{0.8}$

Supplementary Fig. 12 shows the simulated dispersion for bulk and Dirac plasmons in $Bi_2Te_{2.2}Se_{0.8}$, calculated following the same procedure described in the main text for Bi_2Se_3, and employing, as physical parameters, the ones commonly adopted for Bi_2Te_3. Specifically, we consider an effective mass[11] $m_{eff} = 0.044\ m_0$, where m_0 is the electron mass in vacuum, and a Fermi velocity $v=0.5\times10^8$ cm/s, which are different from those adopted in Bi_2Se_3 ($m_{eff}= 0.15\ m_0$, $v=0.623\times10^8$ cm/s[14]). The simulated $q(\lambda_p)$ reported in Supplementary Fig. 12 are at least one order of magnitude smaller (larger) than those observed in experiments.

For calculating the dispersion of bulk plasmons, we consider the bulk carrier density previously evaluated for similar stoichiometry[11] $n_{bv}= 3\times10^{18}$ cm^{-3}. The total carrier density n_M is then evaluated by considering a square-root dependence on the flake thickness d as $n_M = n_s + C\cdot n_{bv} \cdot d^{1/2}$ (Ref.15). Sheet carrier density is taken as $n_s = 2\times10^{12}$ cm^{-2}, estimated by multiplying n_{bv} from Ref. 11 for the thickness of the flake used for the n_{bv} determination (6 QL~8nm).

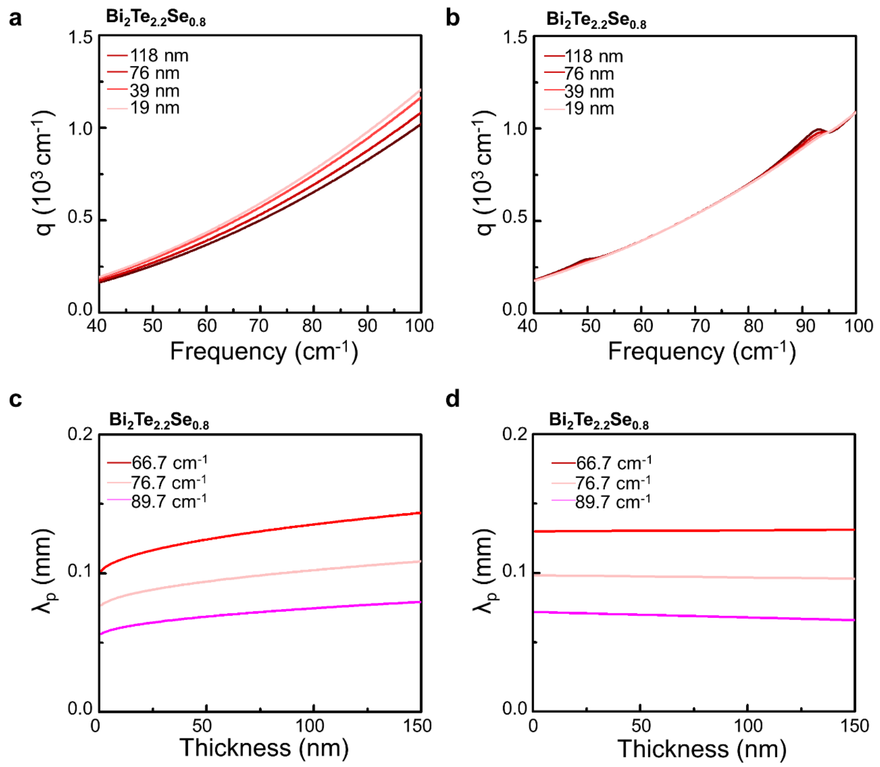

Supplementary Figure 12: Plasmon dispersion. a-b Predicted plasmon energy dispersions in $Bi_2Te_{2.2}Se_{0.8}$, calculated using the physical parameters measured experimentally[9] in $Bi_2Te_{2.4}Se_{0.6}$, for flakes of various thickness ranging from 118 nm to 19 nm for massive bulk plasmons (a) and Dirac plasmons (b); **c-d** predicted plasmon wavelength $\lambda_p = 2\pi/q$ calculated using the q values of panels (a-b), for massive bulk plasmons (c) and Dirac plasmons (d).

4.5 Bulk dielectric permittivities of Bi_2Se_3 and Bi_2Te_3

The two material systems investigated in the present work are modeled as anisotropic Drude-Lorentz materials with two different bulk permittivity functions for the in-plane directions $\varepsilon^{\perp}(\omega)$, parallel to the basal plane, and the out-of-plane direction $\varepsilon^{//}(\omega)$, along the c-axis. The giant anisotropy of the crystal structure and of the vibrational properties gives rise to a strong anisotropy in their dielectric response of the two investigated TIs. Each of the two permittivity components is here described by two oscillators corresponding to the α- and β- phonons, while the electronic contribution in the frequency range of interest, which is well-below the electronic gap ($\omega < E_g/\hbar \sim 2400$ cm^{-1} in Bi_2S_3), is described by ε_∞.

$$\varepsilon^{\perp}(\omega) = \varepsilon_{\infty}^{\perp} + \frac{(s_{\alpha}^{\perp})^2}{\omega_{\alpha}^{\perp 2} - \omega^2 - i\omega\gamma_{\alpha}^{\perp}} + \frac{(s_{\beta}^{\perp})^2}{\omega_{\beta}^{\perp 2} - \omega^2 - i\omega\gamma_{\beta}^{z}} \; \varepsilon^{//}(\omega) = \varepsilon_{\infty}^{z} + \frac{(s_{\alpha}^{z})^2}{\omega_{\alpha}^{z 2} - \omega^2 - i\omega\gamma_{\alpha}^{z}} + \frac{(s_{\beta}^{z})^2}{\omega_{\beta}^{z 2} - \omega^2 - i\omega\gamma_{\beta}^{z}} \qquad \text{Eq. (1)}$$

In Supplementary Table 1 and Table 2 we report the parameters used to model Bi_2Se_3 and Bi_2Te_3 respectively, chosen from available experimental and theoretical literature[16–18].

	ε_∞	$s_\alpha(cm^{-1})$	$s_\beta(cm^{-1})$	$\omega_\alpha(cm^{-1})$	$\omega_\beta(cm^{-1})$	$\gamma_\alpha(cm^{-1})$	$\gamma_\beta(cm^{-1})$
⊥	29	704	55	64	125	3.5	3.5
//	17.4	283	156	135	156	3.5	3.5

Supplementary Table 1. Frequency of the α and β phonon modes ω_x^y, their strength s_x^y and the losses γ_x^y for Bi_2Se_3 from Ref.16 of the main text.

	ε_∞	$s_\alpha(cm^{-1})$	$s_\beta(cm^{-1})$	$\omega_\alpha(cm^{-1})$	$\omega_\beta(cm^{-1})$	$\gamma_\alpha(cm^{-1})$	$\gamma_\beta(cm^{-1})$
⊥	85	667	181	50	95	3.5	10
//	50	314	353	94	120	3.5	15

Supplementary Table 2. Frequency of the α and β phonon modes ω_x^y, their strength s_x^y and the losses γ_x^y for Bi_2Te_3.

The calculated bulk permittivities are displayed in Supplementary Fig. 13. They show two hyperbolic bands in the frequency range 50-200 cm^{-1} where the real parts of $\varepsilon^{\perp}$ and $\varepsilon^{//}$ have opposite signs. The different chemical composition results into a red-shift and sharpening of the hyperbolic bands in Bi_2Te_3 compared to Bi_2Se_3. Both materials also include a Reststrahlen band where both the permittivity components assume negative values. The frequency range investigated in our work addresses the region of type II (yellow shadows in Supplementary Fig. 12) hyperbolicity of the two materials.

The near-field scattering signal expected for bulk Bi_2Se_3 and Bi_2Te_3 reported in Supplementary Fig.5a has been obtained considering the bulk permittivity in Supplementary Fig. 13 and applying the model for the near-field interaction[17].

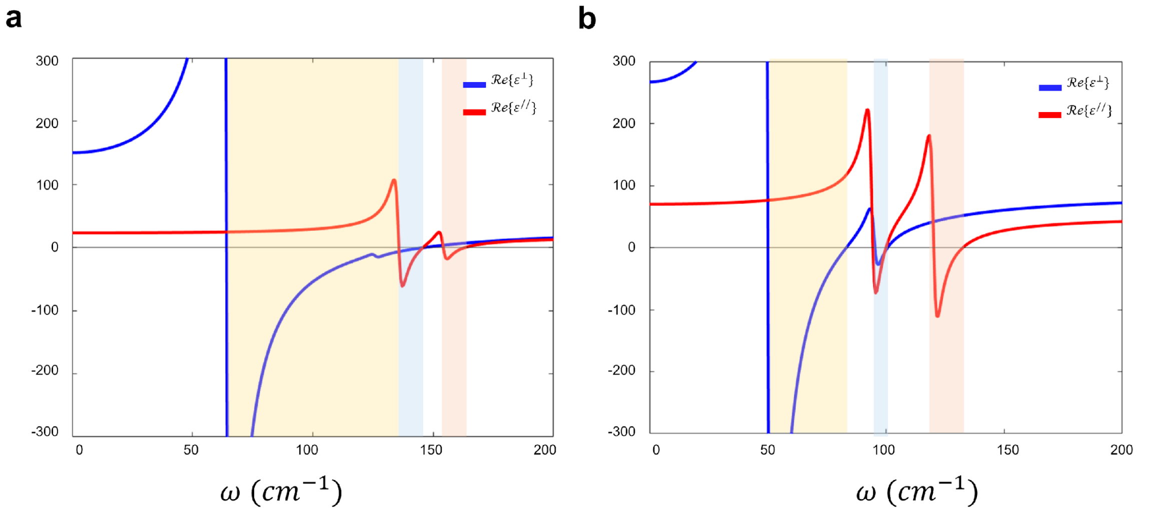

Supplementary Figure 13: Bulk permittivity. a-b In-plane and out-of-plane bulk permittivities of Bi_2Se_3 (a) and Bi_2Te_3 (b) calculated using Eq.1 and parameters of Table 1 (a) and Table 2 (b) with γ_x^y= 3.5 cm^{-1}. Hyperbolic bands of type II ($\mathrm{Re}(\varepsilon^{\perp}) < 0, \mathrm{Re}(\varepsilon^{//}) > 0$) and type I ($\mathrm{Re}(\varepsilon^{\perp}) > 0, \mathrm{Re}(\varepsilon^{//}) < 0$) are outlined as yellow and orange shaded regions, respectively, while the Reststrahlen band ($\mathrm{Re}(\varepsilon^{//}) < 0, \mathrm{Re}(\varepsilon^{z}) < 0$) is indicated by the blue shaded region.

4.6 Hybrid modes in $Bi_2Te_{2.2}Se_{0.8}$

We evaluate the dispersion of hybrid phonon-polariton collective modes for thin flakes of Bi_2Te_3 in the case of zero and finite doping (Supplementary Fig. 14). The dispersions of the hyperbolic bands, in the frequency region of interest, appear broadened and blue-shifted when a finite doping is included $\mu > 0$, compared to the case $\mu=0$ when Dirac plasmons cannot exist.

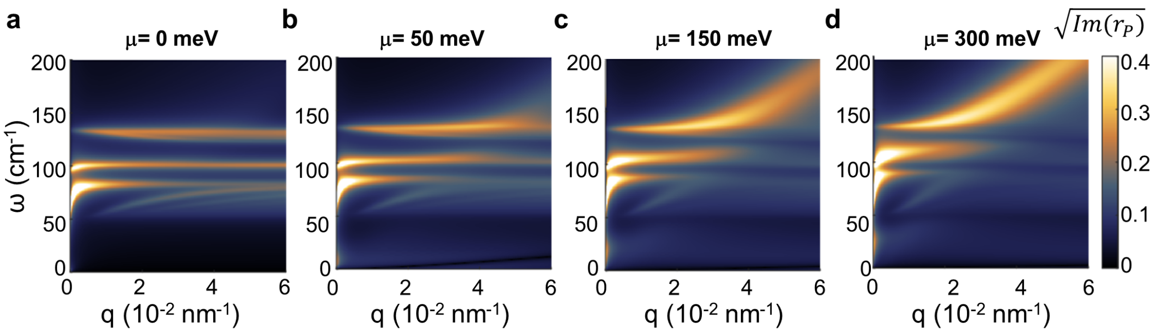

Supplementary Figure 14: Collective modes of Bi_2Te_3. a-b Collective modes dispersion of a Bi_2Te_3 slab of thickness d = 118 nm rendered using the false color maps of Im (r_P) with zero doping (a), when no Dirac plasmons exist and the collective modes are limited to hyperbolic phonon-polaritons (HP^2) and (b-d) with chemical potential μ = 50, 150, 250 meV. Inside the band $\omega_{to}^{\perp} < \omega < \omega_{to}^{//}$, multiple branches of HP^3 are formed due to hybridization of the Dirac plasmons with the HP^2 waveguide modes.

These modifications can be ascribed to the hybridization of the hyperbolic phonons with Dirac plasmons, giving rise to hyperbolic plasmon-phonon-polaritons (HP^3) modes previously predicted for Bi_2Se_3[16].

4.7 Simulation of the near-field signal s_3

We model the tip as a metallic spheroid with curvature radius r= 20 nm and length l= 80 μm considering the scattering problem in the quasi-static approximation[16,18]. We compute the full scattering signal s for a set of different tip heights z_{tip}, defined with respect to the sample surface. The tapping is modelled as an oscillation of z_{tip} with amplitude Δz= 160 nm with minimum tip-sample distance z_{min}= 10 nm. We calculate *s* as the product of the tip-sample polarizability $\chi^{\perp}$ (ω, z_{tip}) and the far-field factor F= $|1+r_p|^2$. The far-field factor accounts for the fact that the source and the detector are in the far-field[18-20] and includes the reflectivity r_p evaluated at q= c/ω. The third-order near-field signal s_3 corresponds to the third Fourier harmonic of s(z_{tip}). The third order polarizability $\chi_3^{\perp}$(ω, z_{tip}) depends both on the geometrical properties of the tip and on the sample reflectivity r_p, and, as a consequence of the finite q-dependence of r_p of the TI, it is momentum

dependent. Specifically, we compute the k residues $R_k^{\perp}$ and poles $\beta_k^{\perp}$ of the polarizability $\chi_3^{\perp}$ $(\omega, z_{tip}) = \sum_{k=0}^{\infty} \frac{R_k^{\perp}}{\beta_k^{\perp} - r_p}$ as a function of z_{tip}, following the generalized spectral method for spheroidal tips[18] including k= 200 terms, and using an effective reflectivity averaged within the momentum range from 0 up to 3 nm^{-1}, weighting the different *q* with normalized Gaussian distribution functions centered at $1/z_{tip.}$ The retrieved signal for a slab of Bi_2Te_3 with chemical potential μ= 200 meV (i.e. below the bandgap of $Bi_2Te_{2.2}\,Se_{0.8}$) is reported in Supplementary Fig. 15 after dividing for the signal expected from the SiO_2/Si substrate modelled by constant permittivity ε_{sub}= 6.3. We observe a peak in s_3 at the frequency of the phonon mode $\omega_\alpha^{\perp}$= 50 cm^{-1} (see Supplementary Note 4.5 for bulk phonon modelling) which is the analogue of the s_3 peak at 64 cm^{-1} previously predicted for Bi_2Se_3[16]. This peak originates from the far-field factor that has a maximum at $\omega_\alpha^{\perp}$. Conversely, the peak centered at 100 cm^{-1} can be attributed to surface modes: hyperbolic phonon-polaritons that hybridize with Dirac plasmons. The coupling with the tip, included in the tip-sample polarizability, results into a small (few cm^{-1}) difference between the frequency position of this peak in s_3 and the corresponding peak in the sample reflectivity Im (r_p) at $\omega_\alpha^{//}$= 95 cm^{-1}. The simulated trend accounts for the contrast change in Fig. 3d when passing from ω= 66.7 cm^{-1} to 76.7 cm^{-1}, and predicts the signal increase observed at 89.7 cm^{-1} as approaching the expected peak centered at 100 cm^{-1} and the stronger thickness dependence at 66.7 cm^{-1} compared to 89.7 cm^{-1} that results from the decrease of r_p with thickness. Finally, the signal phase is expected to increase approaching the peak in the amplitude at 100 cm^{-1} and could be compatible with the phase variation observed at 90 cm^{-1} in the experiments, see Supplementary Fig. 9, after considering a rigid shift to lower frequency of the predicted trend, shown in Fig. Supplementary Fig. 15b.

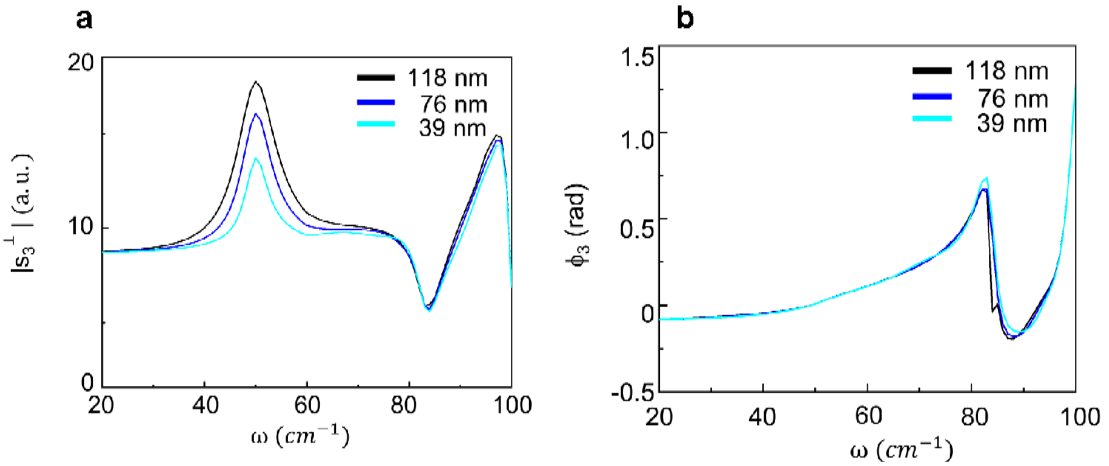

Supplementary Figure 15: Simulation of the s-SNOM signal for a Bi_2Te_3 slab. a-b Expected amplitude (a) and (b) phase of the signal, considering μ= 200 meV and thickness d= {118, 76, 39} nm (colored solid line) referenced to the s_3 signal expected for a material with ε_{sub}= 6.3 that mimics the dielectric response of the SiO_2/Si substrate.

REFERENCES

1. Teweldebrhan, D., Goyal, V. & Balandin, A. A. Exfoliation and characterization of bismuth telluride atomic quintuples and quasi-two-dimensional crystals. *Nano Lett.* **10**, 1209–1218 (2010).
2. Zhang, J. *et al.* Raman spectroscopy of few-quintuple layer topological insulator Bi_2Se_3 nanoplatelets. *Nano Lett.* **11**, 2407–2414 (2011).
3. Knoll, B. & Keilmann, F. Enhanced dielectric contrast in scattering-type scanning near-field optical microscopy. *Opt. Commun.* **182**, 321–328 (2000).
4. Cvitkovic, A., Ocelic, N. & Hillenbrand, R. Analytical model for quantitative prediction of material contrasts in scattering-type near-field optical microscopy. *Opt. Express* **15**, 8550–8565 (2007).
5. Schneider, S. C., Grafström, S. & Eng, L. M. Scattering near-field optical microscopy of optically anisotropic systems. *Phys. Rev. B - Condens. Matter Mater. Phys.* **71**, 115418 (2005).
6. Politano, A. *et al.* Interplay of surface and Dirac plasmons in topological insulators: The case of Bi_2Se_3. *Phys. Rev. Lett.* **115**, 216802 (2015).
7. Ocelic, N. (2007) Quantitative Near-Field Phonon Polariton Spectroscopy. PhD Thesis, Technische Universitat, Munich, Germany

8. Engelbrecht F. & Helbig R. Effect of crystal anisotropy on the infrared reflectivity of 6H-SiC, *Phys. Rev. B* **48**, 15698-15707 (1993).
9. Mooshammer, F. *et al.* Nanoscale Near-Field Tomography of Surface States on $(Bi_{0.5}Sb_{0.5})_2Te_3$. *Nano Lett.* **18**, 7515–7523 (2018).
10. McLeod, A. S. *et al.* Model for quantitative tip-enhanced spectroscopy and the extraction of nanoscale-resolved optical constants. *Phys. Rev. B - Condens. Matter Mater. Phys.* **90**, 085136 (2014).
11. Shikin, A. M. *et al.* Electronic and spin structure of the topological insulator $Bi_2Te_{2.4}Se_{0.6}$. *Phys. Rev. B - Condens. Matter Mater. Phys.* **89**, 125416 (2014).
12. Viti, L. *et al.* Plasma-wave terahertz detection mediated by topological insulators surface states. *Nano Lett.* **16**, 80–87 (2016).
13. Wang, G. & Cagin, T. Investigation of effective mass of carriers in Bi_2Te_3/Sb_2Te_3 superlattices via electronic structure studies on its component crystals. *Appl. Phys. Lett.* **89**, 152101 (2006).
14. Cao, Y. *et al.* Mapping the orbital wavefunction of the surface states in three-dimensional topological insulators. *Nat. Phys.* **9**, 499–504 (2013).
15. Kim, Y. S. *et al.* Thickness-dependent bulk properties and weak antilocalization effect in topological insulator Bi_2Se_3. *Phys. Rev. B - Condens. Matter Mater. Phys.* **84**, 073109 (2011).
16. Ginley, T. P. & Law, S. Coupled Dirac plasmons in topological insulators. *Adv. Opt. Mater.* **6**, 1800113 (2018).
17. Deshko, Y., Krusin-Elbaum, L., Menon, V., Khanikaev, A. & Trevino, J. Surface plasmon polaritons in topological insulator nano-films and superlattices. *Opt. Express* **24**, 7398 (2016).
18. Wu, J. S., Basov, D. N. & Fogler, M. M. Topological insulators are tunable waveguides for hyperbolic polaritons. *Phys. Rev. B - Condens. Matter Mater. Phys.* **92**, 205430 (2015).
19. Giordano, M. C. *et al.* Phase-resolved terahertz self-detection near-field microscopy. *Opt. Express* **26**, 18423–18435 (2018).
20. Jiang, B. Y., Zhang, L. M., Castro Neto, A. H., Basov, D. N. & Fogler, M. M. Generalized spectral method for near-field optical microscopy. *J. Appl. Phys.* **119**, 054305 (2016).
